# Observation of the ${{{\Lambda }} _{\text {b}}^{{0}}} \rightarrow {{\text {J}/\uppsi }} {{{\Xi }} ^{{-}}} {{\text {K}} ^{{+}}}$ decay

**DOI:** 10.1140/epjc/s10052-024-13114-9

**Published:** 2024-10-15

**Authors:** CMS Collaboration, A. Hayrapetyan, A. Tumasyan, W. Adam, J. W. Andrejkovic, T. Bergauer, S. Chatterjee, K. Damanakis, M. Dragicevic, A. Escalante Del Valle, P. S. Hussain, M. Jeitler, N. Krammer, D. Liko, I. Mikulec, J. Schieck, R. Schöfbeck, D. Schwarz, M. Sonawane, S. Templ, W. Waltenberger, C.-E. Wulz, M. R. Darwish, T. Janssen, P. Van Mechelen, E. S. Bols, J. D’Hondt, S. Dansana, A. De Moor, M. Delcourt, H. El Faham, S. Lowette, I. Makarenko, D. Müller, A. R. Sahasransu, S. Tavernier, M. Tytgat, S. Van Putte, D. Vannerom, B. Clerbaux, G. De Lentdecker, L. Favart, D. Hohov, J. Jaramillo, A. Khalilzadeh, K. Lee, M. Mahdavikhorrami, A. Malara, S. Paredes, L. Pétré, N. Postiau, L. Thomas, M. Vanden Bemden, C. Vander Velde, P. Vanlaer, M. De Coen, D. Dobur, Y. Hong, J. Knolle, L. Lambrecht, G. Mestdach, C. Rendón, A. Samalan, K. Skovpen, N. Van Den Bossche, L. Wezenbeek, A. Benecke, G. Bruno, C. Caputo, C. Delaere, I. S. Donertas, A. Giammanco, K. Jaffel, Sa. Jain, V. Lemaitre, J. Lidrych, P. Mastrapasqua, K. Mondal, T. T. Tran, S. Wertz, G. A. Alves, E. Coelho, C. Hensel, T. Menezes De Oliveira, A. Moraes, P. Rebello Teles, M. Soeiro, W. L. Aldá Júnior, M. Alves Gallo Pereira, M. Barroso Ferreira Filho, H. Brandao Malbouisson, W. Carvalho, J. Chinellato, E. M. Da Costa, G. G. Da Silveira, D. De Jesus Damiao, S. Fonseca De Souza, J. Martins, C. Mora Herrera, K. Mota Amarilo, L. Mundim, H. Nogima, A. Santoro, A. Sznajder, M. Thiel, A. Vilela Pereira, C. A. Bernardes, L. Calligaris, T. R. Fernandez Perez Tomei, E. M. Gregores, P. G. Mercadante, S. F. Novaes, B. Orzari, Sandra S. Padula, A. Aleksandrov, G. Antchev, R. Hadjiiska, P. Iaydjiev, M. Misheva, M. Shopova, G. Sultanov, A. Dimitrov, T. Ivanov, L. Litov, B. Pavlov, P. Petkov, A. Petrov, E. Shumka, S. Keshri, S. Thakur, T. Cheng, Q. Guo, T. Javaid, M. Mittal, L. Yuan, G. Bauer, Z. Hu, J. Liu, K. Yi, G. M. Chen, H. S. Chen, M. Chen, F. Iemmi, C. H. Jiang, A. Kapoor, H. Liao, Z.-A. Liu, F. Monti, M. A. Shahzad, R. Sharma, J. N. Song, J. Tao, C. Wang, J. Wang, Z. Wang, H. Zhang, A. Agapitos, Y. Ban, A. Levin, C. Li, Q. Li, Y. Mao, S. J. Qian, X. Sun, D. Wang, H. Yang, L. Zhang, C. Zhou, Z. You, N. Lu, X. Gao, D. Leggat, H. Okawa, Y. Zhang, Z. Lin, C. Lu, M. Xiao, C. Avila, D. A. Barbosa Trujillo, A. Cabrera, C. Florez, J. Fraga, J. A. Reyes Vega, J. Mejia Guisao, F. Ramirez, M. Rodriguez, J. D. Ruiz Alvarez, D. Giljanovic, N. Godinovic, D. Lelas, A. Sculac, M. Kovac, T. Sculac, P. Bargassa, V. Brigljevic, B. K. Chitroda, D. Ferencek, S. Mishra, A. Starodumov, T. Susa, A. Attikis, K. Christoforou, S. Konstantinou, J. Mousa, C. Nicolaou, F. Ptochos, P. A. Razis, H. Rykaczewski, H. Saka, A. Stepennov, M. Finger, M. Finger, A. Kveton, E. Ayala, E. Carrera Jarrin, H. Abdalla, Y. Assran, M. Abdullah Al-Mashad, M. A. Mahmoud, R. K. Dewanjee, K. Ehataht, M. Kadastik, T. Lange, S. Nandan, C. Nielsen, J. Pata, M. Raidal, L. Tani, C. Veelken, H. Kirschenmann, K. Osterberg, M. Voutilainen, S. Bharthuar, E. Brücken, F. Garcia, J. Havukainen, K. T. S. Kallonen, M. S. Kim, R. Kinnunen, T. Lampén, K. Lassila-Perini, S. Lehti, T. Lindén, M. Lotti, L. Martikainen, M. Myllymäki, M. M. Rantanen, H. Siikonen, E. Tuominen, J. Tuominiemi, P. Luukka, H. Petrow, T. Tuuva, M. Besancon, F. Couderc, M. Dejardin, D. Denegri, J. L. Faure, F. Ferri, S. Ganjour, P. Gras, G. Hamel de Monchenault, V. Lohezic, J. Malcles, J. Rander, A. Rosowsky, M. Ö. Sahin, A. Savoy-Navarro, P. Simkina, M. Titov, M. Tornago, C. Baldenegro Barrera, F. Beaudette, A. Buchot Perraguin, P. Busson, A. Cappati, C. Charlot, F. Damas, O. Davignon, A. De Wit, G. Falmagne, B. A. Fontana Santos Alves, S. Ghosh, A. Gilbert, R. Granier de Cassagnac, A. Hakimi, B. Harikrishnan, L. Kalipoliti, G. Liu, J. Motta, M. Nguyen, C. Ochando, L. Portales, R. Salerno, U. Sarkar, J. B. Sauvan, Y. Sirois, A. Tarabini, E. Vernazza, A. Zabi, A. Zghiche, J.-L. Agram, J. Andrea, D. Apparu, D. Bloch, J.-M. Brom, E. C. Chabert, C. Collard, S. Falke, U. Goerlach, C. Grimault, R. Haeberle, A.-C. Le Bihan, M. A. Sessini, P. Van Hove, S. Beauceron, B. Blancon, G. Boudoul, N. Chanon, J. Choi, D. Contardo, P. Depasse, C. Dozen, H. El Mamouni, J. Fay, S. Gascon, M. Gouzevitch, C. Greenberg, G. Grenier, B. Ille, I. B. Laktineh, M. Lethuillier, L. Mirabito, S. Perries, A. Purohit, M. Vander Donckt, P. Verdier, J. Xiao, G. Adamov, I. Lomidze, Z. Tsamalaidze, V. Botta, L. Feld, K. Klein, M. Lipinski, D. Meuser, A. Pauls, N. Röwert, M. Teroerde, S. Diekmann, A. Dodonova, N. Eich, D. Eliseev, F. Engelke, M. Erdmann, P. Fackeldey, B. Fischer, T. Hebbeker, K. Hoepfner, F. Ivone, A. Jung, M. Y. Lee, L. Mastrolorenzo, M. Merschmeyer, A. Meyer, S. Mukherjee, D. Noll, A. Novak, F. Nowotny, A. Pozdnyakov, Y. Rath, W. Redjeb, F. Rehm, H. Reithler, V. Sarkisovi, A. Schmidt, A. Sharma, A. Stein, F. Torres Da Silva De Araujo, L. Vigilante, S. Wiedenbeck, S. Zaleski, C. Dziwok, G. Flügge, W. Haj Ahmad, T. Kress, A. Nowack, O. Pooth, A. Stahl, T. Ziemons, A. Zotz, H. Aarup Petersen, M. Aldaya Martin, J. Alimena, S. Amoroso, Y. An, S. Baxter, M. Bayatmakou, H. Becerril Gonzalez, O. Behnke, A. Belvedere, S. Bhattacharya, F. Blekman, K. Borras, D. Brunner, A. Campbell, A. Cardini, C. Cheng, F. Colombina, S. Consuegra Rodríguez, G. Correia Silva, M. De Silva, G. Eckerlin, D. Eckstein, L. I. Estevez Banos, O. Filatov, E. Gallo, A. Geiser, A. Giraldi, G. Greau, V. Guglielmi, M. Guthoff, A. Hinzmann, A. Jafari, L. Jeppe, N. Z. Jomhari, B. Kaech, M. Kasemann, H. Kaveh, C. Kleinwort, R. Kogler, M. Komm, D. Krücker, W. Lange, D. Leyva Pernia, K. Lipka, W. Lohmann, R. Mankel, I.-A. Melzer-Pellmann, M. Mendizabal Morentin, J. Metwally, A. B. Meyer, G. Milella, A. Mussgiller, A. Nürnberg, Y. Otarid, D. Pérez Adán, E. Ranken, A. Raspereza, B. Ribeiro Lopes, J. Rübenach, A. Saggio, M. Scham, S. Schnake, P. Schütze, C. Schwanenberger, D. Selivanova, M. Shchedrolosiev, R. E. Sosa Ricardo, L. P. Sreelatha Pramod, D. Stafford, F. Vazzoler, A. Ventura Barroso, R. Walsh, Q. Wang, Y. Wen, K. Wichmann, L. Wiens, C. Wissing, S. Wuchterl, Y. Yang, A. Zimermmane Castro Santos, A. Albrecht, S. Albrecht, M. Antonello, S. Bein, L. Benato, M. Bonanomi, P. Connor, M. Eich, K. El Morabit, Y. Fischer, A. Fröhlich, C. Garbers, E. Garutti, A. Grohsjean, M. Hajheidari, J. Haller, H. R. Jabusch, G. Kasieczka, P. Keicher, R. Klanner, W. Korcari, T. Kramer, V. Kutzner, F. Labe, J. Lange, A. Lobanov, C. Matthies, A. Mehta, L. Moureaux, M. Mrowietz, A. Nigamova, Y. Nissan, A. Paasch, K. J. Pena Rodriguez, T. Quadfasel, B. Raciti, M. Rieger, D. Savoiu, J. Schindler, P. Schleper, M. Schröder, J. Schwandt, M. Sommerhalder, H. Stadie, G. Steinbrück, A. Tews, M. Wolf, S. Brommer, M. Burkart, E. Butz, T. Chwalek, A. Dierlamm, A. Droll, N. Faltermann, M. Giffels, A. Gottmann, F. Hartmann, R. Hofsaess, M. Horzela, U. Husemann, M. Klute, R. Koppenhöfer, M. Link, A. Lintuluoto, S. Maier, S. Mitra, M. Mormile, Th. Müller, M. Neukum, M. Oh, G. Quast, K. Rabbertz, B. Regnery, N. Shadskiy, I. Shvetsov, H. J. Simonis, N. Trevisani, R. Ulrich, J. van der Linden, R. F. Von Cube, M. Wassmer, S. Wieland, F. Wittig, R. Wolf, S. Wunsch, X. Zuo, G. Anagnostou, P. Assiouras, G. Daskalakis, A. Kyriakis, A. Papadopoulos, A. Stakia, P. Kontaxakis, G. Melachroinos, A. Panagiotou, I. Papavergou, I. Paraskevas, N. Saoulidou, K. Theofilatos, E. Tziaferi, K. Vellidis, I. Zisopoulos, G. Bakas, T. Chatzistavrou, G. Karapostoli, K. Kousouris, I. Papakrivopoulos, E. Siamarkou, G. Tsipolitis, A. Zacharopoulou, K. Adamidis, I. Bestintzanos, I. Evangelou, C. Foudas, P. Gianneios, C. Kamtsikis, P. Katsoulis, P. Kokkas, P. G. Kosmoglou Kioseoglou, N. Manthos, I. Papadopoulos, J. Strologas, M. Bartók, C. Hajdu, D. Horvath, F. Sikler, V. Veszpremi, M. Csanád, K. Farkas, M. M. A. Gadallah, Á. Kadlecsik, P. Major, K. Mandal, G. Pásztor, A. J. Rádl, G. I. Veres, P. Raics, B. Ujvari, G. Zilizi, G. Bencze, S. Czellar, J. Karancsi, J. Molnar, Z. Szillasi, T. Csorgo, F. Nemes, T. Novak, J. Babbar, S. Bansal, S. B. Beri, V. Bhatnagar, G. Chaudhary, S. Chauhan, N. Dhingra, A. Kaur, A. Kaur, H. Kaur, M. Kaur, S. Kumar, M. Meena, K. Sandeep, T. Sheokand, J. B. Singh, A. Singla, A. Ahmed, A. Bhardwaj, A. Chhetri, B. C. Choudhary, A. Kumar, M. Naimuddin, K. Ranjan, S. Saumya, S. Acharya, S. Baradia, S. Barman, S. Bhattacharya, D. Bhowmik, S. Dutta, S. Dutta, B. Gomber, P. Palit, G. Saha, B. Sahu, S. Sarkar, M. M. Ameen, P. K. Behera, S. C. Behera, S. Chatterjee, P. Jana, P. Kalbhor, J. R. Komaragiri, D. Kumar, L. Panwar, R. Pradhan, P. R. Pujahari, N. R. Saha, A. Sharma, A. K. Sikdar, S. Verma, T. Aziz, I. Das, S. Dugad, M. Kumar, G. B. Mohanty, P. Suryadevara, A. Bala, S. Banerjee, R. M. Chatterjee, M. Guchait, Sh. Jain, S. Karmakar, S. Kumar, G. Majumder, K. Mazumdar, S. Mukherjee, S. Parolia, A. Thachayath, S. Bahinipati, A. K. Das, C. Kar, D. Maity, P. Mal, T. Mishra, V. K. Muraleedharan Nair Bindhu, K. Naskar, A. Nayak, P. Sadangi, P. Saha, S. K. Swain, S. Varghese, D. Vats, A. Alpana, S. Dube, B. Kansal, A. Laha, A. Rastogi, S. Sharma, H. Bakhshiansohi, E. Khazaie, M. Zeinali, S. Chenarani, S. M. Etesami, M. Khakzad, M. Mohammadi Najafabadi, M. Grunewald, M. Abbrescia, R. Aly, A. Colaleo, D. Creanza, B. D’Anzi, N. De Filippis, M. De Palma, A. Di Florio, W. Elmetenawee, L. Fiore, G. Iaselli, G. Maggi, M. Maggi, I. Margjeka, V. Mastrapasqua, S. My, S. Nuzzo, A. Pellecchia, A. Pompili, G. Pugliese, R. Radogna, G. Ramirez-Sanchez, D. Ramos, A. Ranieri, L. Silvestris, F. M. Simone, Ü. Sözbilir, A. Stamerra, R. Venditti, P. Verwilligen, A. Zaza, G. Abbiendi, C. Battilana, D. Bonacorsi, L. Borgonovi, R. Campanini, P. Capiluppi, F. R. Cavallo, M. Cuffiani, G. M. Dallavalle, T. Diotalevi, F. Fabbri, A. Fanfani, D. Fasanella, P. Giacomelli, L. Giommi, C. Grandi, L. Guiducci, S. Lo Meo, L. Lunerti, S. Marcellini, G. Masetti, F. L. Navarria, A. Perrotta, F. Primavera, A. M. Rossi, T. Rovelli, G. P. Siroli, S. Costa, A. Di Mattia, R. Potenza, A. Tricomi, C. Tuve, G. Barbagli, G. Bardelli, B. Camaiani, A. Cassese, R. Ceccarelli, V. Ciulli, C. Civinini, R. D’Alessandro, E. Focardi, T. Kello, G. Latino, P. Lenzi, M. Lizzo, M. Meschini, S. Paoletti, A. Papanastassiou, G. Sguazzoni, L. Viliani, L. Benussi, S. Bianco, S. Meola, D. Piccolo, P. Chatagnon, F. Ferro, E. Robutti, S. Tosi, A. Benaglia, G. Boldrini, F. Brivio, F. Cetorelli, F. De Guio, M. E. Dinardo, P. Dini, S. Gennai, R. Gerosa, A. Ghezzi, P. Govoni, L. Guzzi, M. T. Lucchini, M. Malberti, S. Malvezzi, A. Massironi, D. Menasce, L. Moroni, M. Paganoni, D. Pedrini, B. S. Pinolini, S. Ragazzi, T. Tabarelli de Fatis, D. Zuolo, S. Buontempo, A. Cagnotta, F. Carnevali, N. Cavallo, A. De Iorio, F. Fabozzi, A. O. M. Iorio, L. Lista, P. Paolucci, B. Rossi, C. Sciacca, R. Ardino, P. Azzi, N. Bacchetta, D. Bisello, P. Bortignon, A. Bragagnolo, R. Carlin, T. Dorigo, F. Gasparinis, U. Gasparini, A. Gozzelino, G. Grosso, L. Layer, E. Lusiani, M. Margoni, A. T. Meneguzzo, M. Migliorini, J. Pazzini, P. Ronchese, R. Rossin, F. Simonetto, G. Strong, M. Tosi, A. Triossi, S. Ventura, H. Yarar, M. Zanetti, P. Zotto, A. Zucchetta, G. Zumerle, S. Abu Zeid, C. Aimè, A. Braghieri, S. Calzaferri, D. Fiorina, P. Montagna, V. Re, C. Riccardi, P. Salvini, I. Vai, P. Vitulo, S. Ajmal, P. Asenov, G. M. Bilei, D. Ciangottini, L. Fanò, M. Magherini, G. Mantovani, V. Mariani, M. Menichelli, F. Moscatelli, A. Piccinelli, M. Presilla, A. Rossi, A. Santocchia, D. Spiga, T. Tedeschi, P. Azzurri, G. Bagliesi, R. Bhattacharya, L. Bianchini, T. Boccali, E. Bossini, D. Bruschini, R. Castaldi, M. A. Ciocci, M. Cipriani, V. D’Amantes, R. Dell’Orso, S. Donato, A. Giassi, F. Ligabue, D. Matos Figueiredo, A. Messineo, M. Musich, F. Palla, A. Rizzi, G. Rolandi, S. Roy Chowdhury, T. Sarkar, A. Scribano, P. Spagnolo, R. Tenchini, G. Tonelli, N. Turini, A. Venturi, P. G. Verdini, P. Barria, M. Campana, F. Cavallari, L. Cunqueiro Mendez, D. Del Re, E. Di Marco, M. Diemoz, F. Errico, E. Longo, P. Meridiani, J. Mijuskovic, G. Organtini, F. Pandolfi, R. Paramatti, C. Quaranta, S. Rahatlou, C. Rovelli, F. Santanastasio, L. Soffi, N. Amapane, R. Arcidiaconos, S. Argiro, M. Arneodo, N. Bartosik, R. Bellan, A. Bellora, C. Biino, N. Cartiglia, M. Costa, R. Covarelli, N. Demaria, L. Finco, M. Grippo, B. Kiani, F. Legger, F. Luongo, C. Mariotti, S. Maselli, A. Mecca, E. Migliore, M. Monteno, R. Mulargia, M. M. Obertino, G. Ortona, L. Pacher, N. Pastrone, M. Pelliccioni, M. Ruspa, F. Siviero, V. Sola, A. Solano, D. Soldi, A. Staiano, C. Tarricone, D. Trocino, G. Umoret, E. Vlasov, S. Belforte, V. Candelise, M. Casarsa, F. Cossutti, K. De Leo, G. Della Ricca, S. Dogra, J. Hong, C. Huh, B. Kim, D. H. Kim, J. Kim, H. Lee, S. W. Lee, C. S. Moon, Y. D. Oh, M. S. Ryu, S. Sekmen, Y. C. Yang, G. Bak, P. Gwak, H. Kim, D. H. Moon, E. Asilar, D. Kim, T. J. Kim, J. A. Merlin, J. Park, S. Choi, S. Han, B. Hong, K. Lee, K. S. Lee, S. Lee, J. Park, S. K. Park, J. Yoo, J. Goh, H. S. Kim, Y. Kim, S. Lee, J. Almond, J. H. Bhyun, J. Choi, W. Jun, J. Kim, J. S. Kim, S. Ko, H. Kwon, H. Lee, J. Lee, J. Lee, B. H. Oh, S. B. Oh, H. Seo, U. K. Yang, I. Yoon, W. Jang, D. Y. Kang, Y. Kang, S. Kim, B. Ko, J. S. H. Lee, Y. Lee, I. C. Park, Y. Roh, I. J. Watson, S. Yang, S. Ha, H. D. Yoo, M. Choi, M. R. Kim, H. Lee, Y. Lee, I. Yu, T. Beyrouthy, Y. Maghrbi, K. Dreimanis, A. Gaile, G. Pikurs, A. Potrebko, M. Seidel, V. Veckalns, N. R. Strautnieks, M. Ambrozas, A. Juodagalvis, A. Rinkevicius, G. Tamulaitis, N. Bin Norjoharuddeen, I. Yusuff, Z. Zolkapli, J. F. Benitez, A. Castaneda Hernandez, H. A. Encinas Acosta, L. G. Gallegos Maríñez, M. León Coello, J. A. Murillo Quijada, A. Sehrawat, L. Valencia Palomo, G. Ayala, H. Castilla-Valdez, E. De La Cruz-Burelo, I. Heredia-De La Cruz, R. Lopez-Fernandez, C. A. Mondragon Herrera, A. Sánchez Hernández, C. Oropeza Barrera, M. Ramírez García, I. Bautista, I. Pedraza, H. A. Salazar Ibarguen, C. Uribe Estrada, I. Bubanja, N. Raicevic, P. H. Butler, A. Ahmad, M. I. Asghar, A. Awais, M. I. M. Awan, H. R. Hoorani, W. A. Khan, V. Avati, L. Grzanka, M. Malawski, H. Bialkowska, M. Bluj, B. Boimska, M. Górski, M. Kazana, M. Szleper, P. Zalewski, K. Bunkowski, K. Doroba, A. Kalinowski, M. Konecki, J. Krolikowski, A. Muhammad, K. Pozniak, W. Zabolotny, M. Araujo, D. Bastos, C. Beirão Da Cruz E Silva, A. Boletti, M. Bozzo, P. Faccioli, M. Gallinaro, J. Hollar, N. Leonardo, T. Niknejad, A. Petrilli, M. Pisano, J. Seixas, J. Varela, J. W. Wulff, P. Adzic, P. Milenovic, M. Dordevic, J. Milosevic, V. Rekovic, M. Aguilar-Benitez, J. Alcaraz Maestre, Cristina F. Bedoya, M. Cepeda, M. Cerrada, N. Colino, B. De La Cruz, A. Delgado Peris, D. Fernández Del Val, J. P. Fernández Ramos, J. Flix, M. C. Fouz, O. Gonzalez Lopez, S. Goy Lopez, J. M. Hernandez, M. I. Josa, J. León Holgado, D. Moran, C. M. Morcillo Perez, Á. Navarro Tobar, C. Perez Dengra, A. Pérez-Calero Yzquierdo, J. Puerta Pelayo, I. Redondo, D. D. Redondo Ferrero, L. Romero, S. Sánchez Navas, L. Urda Gómez, J. Vazquez Escobar, C. Willmott, J. F. de Trocóniz, B. Alvarez Gonzalez, J. Cuevas, J. Fernandez Menendez, S. Folgueras, I. Gonzalez Caballero, J. R. González Fernández, E. Palencia Cortezon, C. Ramón Álvarez, V. Rodríguez Bouza, A. Soto Rodríguez, A. Trapote, C. Vico Villalba, P. Vischia, S. Bhowmik, S. Blanco Fernández, J. A. Brochero Cifuentes, I. J. Cabrillo, A. Calderon, J. Duarte Campderros, M. Fernandez, C. Fernandez Madrazo, G. Gomez, C. Lasaosa García, C. Martinez Rivero, P. Martinez Ruiz del Arbol, F. Matorras, P. Matorras Cuevas, E. Navarrete Ramos, J. Piedra Gomez, L. Scodellaro, I. Vila, J. M. Vizan Garcia, M. K. Jayananda, B. Kailasapathy, D. U. J. Sonnadara, D. D. C. Wickramarathna, W. G. D. Dharmaratna, K. Liyanage, N. Perera, N. Wickramage, D. Abbaneo, C. Amendola, E. Auffray, G. Auzinger, J. Baechler, D. Barney, A. Bermúdez Martínez, M. Bianco, B. Bilin, A. A. Bin Anuar, A. Bocci, E. Brondolin, C. Caillol, T. Camporesi, G. Cerminara, N. Chernyavskaya, D. d’Enterria, A. Dabrowski, A. David, A. De Roeck, M. M. Defranchis, M. Deile, M. Dobson, F. Fallavollita, L. Forthomme, G. Franzoni, W. Funk, S. Giani, D. Gigi, K. Gill, F. Glege, L. Gouskos, M. Haranko, J. Hegeman, B. Huber, V. Innocente, T. James, P. Janot, J. Kieseler, S. Laurila, P. Lecoq, E. Leutgeb, C. Lourenço, B. Maier, L. Malgeri, M. Mannelli, A. C. Marini, M. Matthewman, F. Meijers, S. Mersi, E. Meschi, V. Milosevic, F. Moortgat, M. Mulders, S. Orfanelli, F. Pantaleo, G. Petrucciani, A. Pfeiffer, M. Pierini, D. Piparo, H. Qu, D. Rabady, G. Reales Gutiérrez, M. Rovere, H. Sakulin, S. Scarfi, C. Schwick, M. Selvaggi, A. Sharma, K. Shchelina, P. Silva, P. Sphicas, A. G. Stahl Leiton, A. Steen, S. Summers, D. Treille, P. Tropea, A. Tsirou, D. Walter, J. Wanczyk, K. A. Wozniak, P. Zehetner, P. Zejdl, W. D. Zeuner, T. Bevilacqua, L. Caminada, A. Ebrahimi, W. Erdmann, R. Horisberger, Q. Ingram, H. C. Kaestli, D. Kotlinski, C. Lange, M. Missiroli, L. Noehte, T. Rohe, T. K. Aarrestad, K. Androsov, M. Backhaus, A. Calandri, C. Cazzaniga, K. Datta, A. De Cosa, G. Dissertori, M. Dittmar, M. Donegà, F. Eble, M. Galli, K. Gedia, F. Glessgen, C. Grab, D. Hits, W. Lustermann, A.-M. Lyon, R. A. Manzoni, M. Marchegiani, L. Marchese, C. Martin Perez, A. Mascellani, F. Nessi-Tedaldi, F. Pauss, V. Perovic, S. Pigazzini, M. G. Ratti, M. Reichmann, C. Reissel, T. Reitenspiess, B. Ristic, F. Riti, D. Ruini, D. A. Sanz Becerra, R. Seidita, J. Steggemann, D. Valsecchi, R. Wallny, C. Amsler, P. Bärtschi, C. Botta, D. Brzhechko, M. F. Canelli, K. Cormier, R. Del Burgo, J. K. Heikkilä, M. Huwiler, W. Jin, A. Jofrehei, B. Kilminster, S. Leontsinis, S. P. Liechti, A. Macchiolo, P. Meiring, V. M. Mikuni, U. Molinatti, I. Neutelings, A. Reimers, P. Robmann, S. Sanchez Cruz, K. Schweiger, M. Senger, Y. Takahashi, R. Tramontano, C. Adloff, C. M. Kuo, W. Lin, P. K. Rout, P. C. Tiwari, S. S. Yu, L. Ceard, Y. Chao, K. F. Chen, P. s. Chen, Z. g. Chen, W.-S. Hou, T. h. Hsu, Y. w. Kao, R. Khurana, G. Kole, Y. Y. Li, R.-S. Lu, E. Paganis, A. Psallidas, X. f. Su, J. Thomas-Wilsker, L. s. Tsai, H. y. Wu, E. Yazgan, C. Asawatangtrakuldee, N. Srimanobhas, V. Wachirapusitanand, D. Agyel, F. Boran, Z. S. Demiroglu, F. Dolek, I. Dumanoglu, E. Eskut, Y. Guler, E. Gurpinar Guler, C. Isik, O. Kara, A. Kayis Topaksu, U. Kiminsu, G. Onengut, K. Ozdemir, A. Polatoz, B. Tali, U. G. Tok, S. Turkcapar, E. Uslan, I. S. Zorbakir, M. Yalvac, B. Akgun, I. O. Atakisi, E. Gülmez, M. Kaya, O. Kaya, S. Tekten, A. Cakir, K. Cankocak, Y. Komurcu, S. Sen, O. Aydilek, S. Cerci, V. Epshteyn, B. Hacisahinoglu, I. Hos, B. Isildak, B. Kaynak, S. Ozkorucuklu, O. Potok, H. Sert, C. Simsek, D. Sunar Cerci, C. Zorbilmez, A. Boyaryntsev, B. Grynyov, L. Levchuk, D. Anthony, J. J. Brooke, A. Bundock, F. Bury, E. Clement, D. Cussans, H. Flacher, M. Glowacki, J. Goldstein, H. F. Heath, L. Kreczko, B. Krikler, S. Paramesvaran, S. Seif El Nasr-Storey, V. J. Smith, N. Stylianou, K. Walkingshaw Pass, R. White, A. H. Ball, K. W. Bell, A. Belyaev, C. Brew, R. M. Brown, D. J. A. Cockerill, C. Cooke, K. V. Ellis, K. Harder, S. Harper, M.-L. Holmberg, J. Linacre, K. Manolopoulos, D. M. Newbold, E. Olaiya, D. Petyt, T. Reis, G. Salvi, T. Schuh, C. H. Shepherd-Themistocleous, I. R. Tomalin, T. Williams, R. Bainbridge, P. Bloch, C. E. Brown, O. Buchmuller, V. Cacchio, C. A. Carrillo Montoya, G. S. Chahal, D. Colling, J. S. Dancu, P. Dauncey, G. Davies, J. Davies, M. Della Negra, S. Fayer, G. Fedi, G. Hall, M. H. Hassanshahi, A. Howard, G. Iles, M. Knight, J. Langford, L. Lyons, A.-M. Magnan, S. Malik, A. Martelli, M. Mieskolainen, J. Nash, M. Pesaresi, B. C. Radburn-Smith, A. Richards, A. Rose, C. Seez, R. Shukla, A. Tapper, K. Uchida, G. P. Uttley, L. H. Vage, T. Virdee, M. Vojinovic, N. Wardle, D. Winterbottom, K. Coldham, J. E. Cole, A. Khan, P. Kyberd, I. D. Reid, S. Abdullin, A. Brinkerhoff, B. Caraway, J. Dittmann, K. Hatakeyama, J. Hiltbrand, A. R. Kanuganti, B. McMaster, M. Saunders, S. Sawant, C. Sutantawibul, M. Toms, J. Wilson, R. Bartek, A. Dominguez, C. Huerta Escamilla, A. E. Simsek, R. Uniyal, A. M. Vargas Hernandez, R. Chudasama, S. I. Cooper, S. V. Gleyzer, C. U. Perez, P. Rumerio, E. Usai, C. West, R. Yi, A. Akpinar, A. Albert, D. Arcaro, C. Cosby, Z. Demiragli, C. Erice, E. Fontanesi, D. Gastler, S. Jeon, J. Rohlf, K. Salyer, D. Sperka, D. Spitzbart, I. Suarez, A. Tsatsos, S. Yuan, G. Benelli, X. Coubez, D. Cutts, M. Hadley, U. Heintz, J. M. Hogan, T. Kwon, G. Landsberg, K. T. Lau, D. Li, J. Luo, S. Mondal, M. Narain, N. Pervan, S. Sagir, F. Simpson, M. Stamenkovic, W. Y. Wong, X. Yan, W. Zhang, S. Abbott, J. Bonilla, C. Brainerd, R. Breedon, M. Calderon De La Barca Sanchez, M. Chertok, M. Citron, J. Conway, P. T. Cox, R. Erbacher, F. Jensen, O. Kukral, G. Mocellin, M. Mulhearn, D. Pellett, W. Wei, Y. Yao, F. Zhang, M. Bachtis, R. Cousins, A. Datta, J. Hauser, M. Ignatenko, M. A. Iqbal, T. Lam, E. Manca, D. Saltzberg, V. Valuev, R. Clare, M. Gordon, G. Hanson, W. Si, S. Wimpenny, J. G. Branson, S. Cittolin, S. Cooperstein, D. Diaz, J. Duarte, L. Giannini, J. Guiang, R. Kansal, V. Krutelyov, R. Lee, J. Letts, M. Masciovecchio, F. Mokhtar, M. Pieri, M. Quinnan, B. V. Sathia Narayanan, V. Sharma, M. Tadel, E. Vourliotis, F. Würthwein, Y. Xiang, A. Yagil, A. Barzdukas, L. Brennan, C. Campagnari, G. Collura, A. Dorsett, J. Incandela, M. Kilpatrick, J. Kim, A. J. Li, P. Masterson, H. Mei, M. Oshiro, J. Richman, U. Sarica, R. Schmitz, F. Setti, J. Sheplock, D. Stuart, S. Wang, A. Bornheim, O. Cerri, A. Latorre, J. M. Lawhorn, J. Mao, H. B. Newman, T. Q. Nguyen, M. Spiropulu, J. R. Vlimant, C. Wang, S. Xie, R. Y. Zhu, J. Alison, S. An, M. B. Andrews, P. Bryant, V. Dutta, T. Ferguson, A. Harilal, C. Liu, T. Mudholkar, S. Murthy, M. Paulini, A. Roberts, A. Sanchez, W. Terrill, J. P. Cumalat, W. T. Ford, A. Hassani, G. Karathanasis, E. MacDonald, N. Manganelli, F. Marini, A. Perloff, C. Savard, N. Schonbeck, K. Stenson, K. A. Ulmer, S. R. Wagner, N. Zipper, J. Alexander, S. Bright-Thonney, X. Chen, D. J. Cranshaw, J. Fan, X. Fan, D. Gadkari, S. Hogan, J. Monroy, J. R. Patterson, J. Reichert, M. Reid, A. Ryd, J. Thom, P. Wittich, R. Zou, M. Albrow, M. Alyari, O. Amram, G. Apollinari, A. Apresyan, L. A. T. Bauerdick, D. Berry, J. Berryhill, P. C. Bhat, K. Burkett, J. N. Butler, A. Canepa, G. B. Cerati, H. W. K. Cheung, F. Chlebana, G. Cummings, J. Dickinson, I. Dutta, V. D. Elvira, Y. Feng, J. Freeman, A. Gandrakota, Z. Gecse, L. Gray, D. Green, A. Grummer, S. Grünendahl, D. Guerrero, O. Gutsche, R. M. Harris, R. Heller, T. C. Herwig, J. Hirschauer, L. Horyn, B. Jayatilaka, S. Jindariani, M. Johnson, U. Joshi, T. Klijnsma, B. Klima, K. H. M. Kwok, S. Lammel, D. Lincoln, R. Lipton, T. Liu, C. Madrid, K. Maeshima, C. Mantilla, D. Mason, P. McBride, P. Merkel, S. Mrenna, S. Nahn, J. Ngadiuba, D. Noonan, V. Papadimitriou, N. Pastika, K. Pedro, C. Pena, F. Ravera, A. Reinsvold Hall, L. Ristori, E. Sexton-Kennedy, N. Smith, A. Soha, L. Spiegel, S. Stoynev, J. Strait, L. Taylor, S. Tkaczyk, N. V. Tran, L. Uplegger, E. W. Vaandering, I. Zoi, C. Aruta, P. Avery, D. Bourilkov, L. Cadamuro, P. Chang, V. Cherepanov, R. D. Field, E. Koenig, M. Kolosova, J. Konigsberg, A. Korytov, K. H. Lo, K. Matchev, N. Menendez, G. Mitselmakher, K. Mohrman, A. Muthirakalayil Madhu, N. Rawal, D. Rosenzweig, S. Rosenzweig, K. Shi, J. Wang, T. Adams, A. Al Kadhim, A. Askew, N. Bower, R. Habibullah, V. Hagopian, R. Hashmi, R. S. Kim, S. Kim, T. Kolberg, G. Martinez, H. Prosper, P. R. Prova, O. Viazlo, M. Wulansatiti, R. Yohay, J. Zhang, B. Alsufyani, M. M. Baarmand, S. Butalla, T. Elkafrawy, M. Hohlmann, R. Kumar Verma, M. Rahmani, M. R. Adams, C. Bennett, R. Cavanaugh, S. Dittmer, R. Escobar Franco, O. Evdokimov, C. E. Gerber, D. J. Hofman, J. H. Lee, D. S. Lemos, A. H. Merrit, C. Mills, S. Nanda, G. Oh, B. Ozek, D. Pilipovic, T. Roy, S. Rudrabhatla, M. B. Tonjes, N. Varelas, X. Wang, Z. Ye, J. Yoo, M. Alhusseini, D. Blend, K. Dilsiz, L. Emediato, G. Karaman, O. K. Köseyan, J.-P. Merlo, A. Mestvirishvili, J. Nachtman, O. Neogi, H. Ogul, Y. Onel, A. Penzo, C. Snyder, E. Tiras, B. Blumenfeld, L. Corcodilos, J. Davis, A. V. Gritsan, L. Kang, S. Kyriacou, P. Maksimovic, M. Roguljic, J. Roskes, S. Sekhar, M. Swartz, T. Á. Vámi, A. Abreu, L. F. Alcerro Alcerro, J. Anguiano, P. Baringer, A. Bean, Z. Flowers, D. Grove, J. King, G. Krintiras, M. Lazarovits, C. Le Mahieu, C. Lindsey, J. Marquez, N. Minafra, M. Murray, M. Nickel, M. Pitt, S. Popescu, C. Rogan, C. Royon, R. Salvatico, S. Sanders, C. Smith, Q. Wang, G. Wilson, B. Allmond, A. Ivanov, K. Kaadze, A. Kalogeropoulos, D. Kim, Y. Maravin, K. Nam, J. Natoli, D. Roy, G. Sorrentino, F. Rebassoo, D. Wright, A. Baden, A. Belloni, A. Bethani, Y. M. Chen, S. C. Eno, N. J. Hadley, S. Jabeen, R. G. Kellogg, T. Koeth, Y. Lai, S. Lascio, A. C. Mignerey, S. Nabili, C. Palmer, C. Papageorgakis, M. M. Paranjpe, L. Wang, J. Bendavid, W. Busza, I. A. Cali, Y. Chen, M. D’Alfonso, J. Eysermans, C. Freer, G. Gomez-Ceballos, M. Goncharov, P. Harris, D. Hoang, D. Kovalskyi, J. Krupa, L. Lavezzo, Y.-J. Lee, K. Long, C. Mironov, C. Paus, D. Rankin, C. Roland, G. Roland, S. Rothman, Z. Shi, G. S. F. Stephans, J. Wang, Z. Wang, B. Wyslouch, T. J. Yang, B. Crossman, B. M. Joshi, C. Kapsiak, M. Krohn, D. Mahon, J. Mans, B. Marzocchi, S. Pandey, M. Revering, R. Rusack, R. Saradhy, N. Schroeder, N. Strobbe, M. A. Wadud, L. M. Cremaldi, K. Bloom, M. Bryson, D. R. Claes, C. Fangmeier, F. Golf, G. Haza, J. Hossain, C. Joo, I. Kravchenko, I. Reed, J. E. Siado, W. Tabb, A. Vagnerini, A. Wightman, F. Yan, D. Yu, A. G. Zecchinelli, G. Agarwal, H. Bandyopadhyay, L. Hay, I. Iashvili, A. Kharchilava, M. Morris, D. Nguyen, S. Rappoccio, H. Rejeb Sfar, A. Williams, E. Barberis, Y. Haddad, Y. Han, A. Krishna, J. Li, M. Lu, G. Madigan, R. Mccarthy, D. M. Morse, V. Nguyen, T. Orimoto, A. Parker, L. Skinnari, A. Tishelman-Charny, B. Wang, D. Wood, S. Bhattacharya, J. Bueghly, Z. Chen, K. A. Hahn, Y. Liu, Y. Miao, D. G. Monk, M. H. Schmitt, A. Taliercio, M. Velasco, R. Band, R. Bucci, S. Castells, M. Cremonesi, A. Das, R. Goldouzian, M. Hildreth, K. W. Ho, K. Hurtado Anampa, C. Jessop, K. Lannon, J. Lawrence, N. Loukas, L. Lutton, J. Mariano, N. Marinelli, I. Mcalister, T. McCauley, C. Mcgrady, C. Moore, Y. Musienko, H. Nelson, M. Osherson, R. Ruchti, A. Townsend, M. Wayne, H. Yockey, M. Zarucki, L. Zygala, A. Basnet, B. Bylsma, M. Carrigan, L. S. Durkin, C. Hill, M. Joyce, A. Lesauvage, M. Nunez Ornelas, K. Wei, B. L. Winer, B. R. Yates, F. M. Addesa, H. Bouchamaoui, P. Das, G. Dezoort, P. Elmer, A. Frankenthal, B. Greenberg, N. Haubrich, S. Higginbotham, G. Kopp, S. Kwan, D. Lange, A. Loeliger, D. Marlow, I. Ojalvo, J. Olsen, A. Shevelev, D. Stickland, C. Tully, S. Malik, A. S. Bakshi, V. E. Barnes, S. Chandra, R. Chawla, S. Das, A. Gu, L. Gutay, M. Jones, A. W. Jung, D. Kondratyev, A. M. Koshy, M. Liu, G. Negro, N. Neumeister, G. Paspalaki, S. Piperov, V. Scheurer, J. F. Schulte, M. Stojanovic, J. Thieman, A. K. Virdi, F. Wang, W. Xie, J. Dolen, N. Parashar, A. Pathak, D. Acosta, A. Baty, T. Carnahan, K. M. Ecklund, P. J. Fernández Manteca, S. Freed, P. Gardner, F. J. M. Geurts, A. Kumar, W. Li, O. Miguel Colin, B. P. Padley, R. Redjimi, J. Rotter, E. Yigitbasi, Y. Zhang, A. Bodek, P. de Barbaro, R. Demina, J. L. Dulemba, C. Fallon, A. Garcia-Bellido, O. Hindrichs, A. Khukhunaishvili, P. Parygin, E. Popova, R. Taus, G. P. Van Onsem, K. Goulianos, B. Chiarito, J. P. Chou, Y. Gershtein, E. Halkiadakis, A. Hart, M. Heindl, D. Jaroslawski, O. Karacheban, I. Laflotte, A. Lath, R. Montalvo, K. Nash, H. Routray, S. Salur, S. Schnetzer, S. Somalwar, R. Stone, S. A. Thayil, S. Thomas, J. Vora, H. Wang, H. Acharya, D. Ally, A. G. Delannoy, S. Fiorendi, T. Holmes, N. Karunarathna, L. Lee, E. Nibigira, S. Spanier, D. Aebi, M. Ahmad, O. Bouhali, M. Dalchenko, R. Eusebi, J. Gilmore, T. Huang, T. Kamon, H. Kim, S. Luo, S. Malhotra, R. Mueller, D. Overton, D. Rathjens, A. Safonov, N. Akchurin, J. Damgov, V. Hegde, A. Hussain, Y. Kazhykarim, K. Lamichhane, S. W. Lee, A. Mankel, T. Mengke, S. Muthumuni, T. Peltola, I. Volobouev, A. Whitbeck, E. Appelt, S. Greene, A. Gurrola, W. Johns, R. Kunnawalkam Elayavalli, A. Melo, F. Romeo, P. Sheldon, S. Tuo, J. Velkovska, J. Viinikainen, B. Cardwell, B. Cox, J. Hakala, R. Hirosky, A. Ledovskoy, A. Li, C. Neu, C. E. Perez Lara, P. E. Karchin, A. Aravind, S. Banerjee, K. Black, T. Bose, S. Dasu, I. De Bruyn, P. Everaerts, C. Galloni, H. He, M. Herndon, A. Herve, C. K. Koraka, A. Lanaro, R. Loveless, J. Madhusudanan Sreekala, A. Mallampalli, A. Mohammadi, S. Mondal, G. Parida, D. Pinna, A. Savin, V. Shang, V. Sharma, W. H. Smith, D. Teague, H. F. Tsoi, W. Vetens, A. Warden, S. Afanasiev, V. Andreev, Yu. Andreev, T. Aushev, M. Azarkin, A. Babaev, A. Belyaev, V. Blinov, E. Boos, V. Borshch, D. Budkouski, V. Chekhovsky, R. Chistov, M. Danilov, A. Dermenev, T. Dimova, D. Druzhkin, M. Dubinin, L. Dudko, A. Ershov, G. Gavrilov, V. Gavrilov, S. Gninenko, V. Golovtcov, N. Golubev, I. Golutvin, I. Gorbunov, A. Gribushin, Y. Ivanov, V. Kachanov, L. Kardapoltsev, V. Karjavine, A. Karneyeu, V. Kim, M. Kirakosyan, D. Kirpichnikov, M. Kirsanov, V. Klyukhin, O. Kodolova, D. Konstantinov, V. Korenkov, A. Kozyrev, N. Krasnikov, A. Lanev, P. Levchenko, N. Lychkovskaya, V. Makarenko, A. Malakhov, V. Matveev, V. Murzin, A. Nikitenko, S. Obraztsov, V. Oreshkin, V. Palichik, V. Perelygin, S. Petrushanko, S. Polikarpov, V. Popov, O. Radchenko, M. Savina, V. Savrin, M. Sergeev, V. Shalaev, S. Shmatov, S. Shulha, Y. Skovpen, S. Slabospitskii, V. Smirnov, A. Snigirev, D. Sosnov, V. Sulimov, E. Tcherniaev, A. Terkulov, O. Teryaev, I. Tlisova, A. Toropin, L. Uvarov, A. Uzunian, A. Vorobyev, N. Voytishin, B. S. Yuldashev, A. Zarubin, I. Zhizhin, A. Zhokin

**Affiliations:** 1 Yerevan Physics Institute, Yerevan, Armenia; 2 Institut für Hochenergiephysik, Vienna, Austria; 3 Universiteit Antwerpen, Antwerpen, Belgium; 4 Vrije Universiteit Brussel, Brussel, Belgium; 5 Université Libre de Bruxelles, Bruxelles, Belgium; 6 Ghent University, Ghent, Belgium; 7 Université Catholique de Louvain, Louvain-la-Neuve, Belgium; 8 Centro Brasileiro de Pesquisas Fisicas, Rio de Janeiro, Brazil; 9 Universidade do Estado do Rio de Janeiro, Rio de Janeiro, Brazil; 10 Universidade Estadual Paulista, Universidade Federal do ABC, São Paulo, Brazil; 11 Institute for Nuclear Research and Nuclear Energy, Bulgarian Academy of Sciences, Sofia, Bulgaria; 12 University of Sofia, Sofia, Bulgaria; 13 Instituto De Alta Investigación, Universidad de Tarapacá, Casilla 7 D, Arica, Chile; 14 Beihang University, Beijing, China; 15 Department of Physics, Tsinghua University, Beijing, China; 16 Institute of High Energy Physics, Beijing, China; 17 State Key Laboratory of Nuclear Physics and Technology, Peking University, Beijing, China; 18 Sun Yat-Sen University, Guangzhou, China; 19 University of Science and Technology of China, Hefei, China; 20 Institute of Modern Physics and Key Laboratory of Nuclear Physics and Ion-beam Application (MOE)-Fudan University, Shanghai, China; 21 Zhejiang University, Hangzhou, Zhejiang China; 22 Universidad de Los Andes, Bogota, Colombia; 23 Universidad de Antioquia, Medellin, Colombia; 24 University of Split, Faculty of Electrical Engineering, Mechanical Engineering and Naval Architecture, Split, Croatia; 25 University of Split, Faculty of Science, Split, Croatia; 26 Institute Rudjer Boskovic, Zagreb, Croatia; 27 University of Cyprus, Nicosia, Cyprus; 28 Charles University, Prague, Czech Republic; 29 Escuela Politecnica Nacional, Quito, Ecuador; 30 Universidad San Francisco de Quito, Quito, Ecuador; 31 Academy of Scientific Research and Technology of the Arab Republic of Egypt, Egyptian Network of High Energy Physics, Cairo, Egypt; 32 Center for High Energy Physics (CHEP-FU), Fayoum University, El-Fayoum, Egypt; 33 National Institute of Chemical Physics and Biophysics, Tallinn, Estonia; 34 Department of Physics, University of Helsinki, Helsinki, Finland; 35 Helsinki Institute of Physics, Helsinki, Finland; 36 Lappeenranta-Lahti University of Technology, Lappeenranta, Finland; 37 IRFU, CEA, Université Paris-Saclay, Gif-sur-Yvette, France; 38 Laboratoire Leprince-Ringuet, CNRS/IN2P3, Ecole Polytechnique, Institut Polytechnique de Paris, Palaiseau, France; 39 Université de Strasbourg, CNRS, IPHC UMR 7178, Strasbourg, France; 40 Institut de Physique des 2 Infinis de Lyon (IP2I ), Villeurbanne, France; 41 Georgian Technical University, Tbilisi, Georgia; 42 RWTH Aachen University, I. Physikalisches Institut, Aachen, Germany; 43 RWTH Aachen University, III. Physikalisches Institut A, Aachen, Germany; 44 RWTH Aachen University, III. Physikalisches Institut B, Aachen, Germany; 45 Deutsches Elektronen-Synchrotron, Hamburg, Germany; 46 University of Hamburg, Hamburg, Germany; 47 Karlsruher Institut fuer Technologie, Karlsruhe, Germany; 48 Institute of Nuclear and Particle Physics (INPP), NCSR Demokritos, Aghia Paraskevi, Greece; 49 National and Kapodistrian University of Athens, Athens, Greece; 50 National Technical University of Athens, Athens, Greece; 51 University of Ioánnina, Ioannina, Greece; 52 HUN-REN Wigner Research Centre for Physics, Budapest, Hungary; 53 MTA-ELTE Lendület CMS Particle and Nuclear Physics Group, Eötvös Loránd University, Budapest, Hungary; 54 Faculty of Informatics, University of Debrecen, Debrecen, Hungary; 55 Institute of Nuclear Research ATOMKI, Debrecen, Hungary; 56 Karoly Robert Campus, MATE Institute of Technology, Gyongyos, Hungary; 57 Panjab University, Chandigarh, India; 58 University of Delhi, Delhi, India; 59 Saha Institute of Nuclear Physics, HBNI, Kolkata, India; 60 Indian Institute of Technology Madras, Madras, India; 61 Tata Institute of Fundamental Research-A, Mumbai, India; 62 Tata Institute of Fundamental Research-B, Mumbai, India; 63 National Institute of Science Education and Research, An OCC of Homi Bhabha National Institute, Bhubaneswar, Odisha India; 64 Indian Institute of Science Education and Research (IISER), Pune, India; 65 Isfahan University of Technology, Isfahan, Iran; 66 Institute for Research in Fundamental Sciences (IPM), Tehran, Iran; 67 University College Dublin, Dublin, Ireland; 68 INFN Sezione di Bari, Università di Bari, Politecnico di Bari, Bari, Italy; 69 INFN Sezione di Bologna, Università di Bologna, Bologna, Italy; 70 INFN Sezione di Catania, Università di Catania, Catania, Italy; 71 INFN Sezione di Firenze, Università di Firenze, Firenze, Italy; 72 INFN Laboratori Nazionali di Frascati, Frascati, Italy; 73 INFN Sezione di Genova, Università di Genova, Genoa, Italy; 74 INFN Sezione di Milano-Bicocca, Università di Milano-Bicocca, Milan, Italy; 75 INFN Sezione di Napoli, Università di Napoli ’Federico II’, Napoli, Italy; Università della Basilicata, Potenza, Italy; Scuola Superiore Meridionale (SSM), Naples, Italy; 76 INFN Sezione di Padova, Università di Padova, Padova, Italy; Università di Trento, Trento, Italy; 77 INFN Sezione di Pavia, Università di Pavia, Pavia, Italy; 78 INFN Sezione di Perugia, Università di Perugia, Perugia, Italy; 79 INFN Sezione di Pisa, Università di Pisa, Scuola Normale Superiore di Pisa, Pisa, Italy; Università di Siena, Siena, Italy; 80 INFN Sezione di Roma, Sapienza Università di Roma, Rome, Italy; 81 INFN Sezione di Torino, Università di Torino, Torino, Italy; Università del Piemonte Orientale, Novara, Italy; 82 INFN Sezione di Trieste, Università di Trieste, Trieste, Italy; 83 Kyungpook National University, Daegu, Korea; 84 Chonnam National University, Institute for Universe and Elementary Particles, Kwangju, Korea; 85 Hanyang University, Seoul, Korea; 86 Korea University, Seoul, Korea; 87 Kyung Hee University, Department of Physics, Seoul, Korea; 88 Sejong University, Seoul, Korea; 89 Seoul National University, Seoul, Korea; 90 University of Seoul, Seoul, Korea; 91 Department of Physics, Yonsei University, Seoul, Korea; 92 Sungkyunkwan University, Suwon, Korea; 93 College of Engineering and Technology, American University of the Middle East (AUM), Dasman, Kuwait; 94 Riga Technical University, Riga, Latvia; 95 University of Latvia (LU), Riga, Latvia; 96 Vilnius University, Vilnius, Lithuania; 97 National Centre for Particle Physics, Universiti Malaya, Kuala Lumpur, Malaysia; 98 Universidad de Sonora (UNISON), Hermosillo, Mexico; 99 Centro de Investigacion y de Estudios Avanzados del IPN, Mexico City, Mexico; 100 Universidad Iberoamericana, Mexico City, Mexico; 101 Benemerita Universidad Autonoma de Puebla, Puebla, Mexico; 102 University of Montenegro, Podgorica, Montenegro; 103 University of Canterbury, Christchurch, New Zealand; 104 National Centre for Physics, Quaid-I-Azam University, Islamabad, Pakistan; 105 AGH University of Krakow, Faculty of Computer Science, Electronics and Telecommunications, Kraków, Poland; 106 National Centre for Nuclear Research, Swierk, Poland; 107 Institute of Experimental Physics, Faculty of Physics, University of Warsaw, Warsaw, Poland; 108 Warsaw University of Technology, Warsaw, Poland; 109 Laboratório de Instrumentação e Física Experimental de Partículas, Lisbon, Portugal; 110 Faculty of Physics, University of Belgrade, Belgrade, Serbia; 111 VINCA Institute of Nuclear Sciences, University of Belgrade, Belgrade, Serbia; 112 Centro de Investigaciones Energéticas Medioambientales y Tecnológicas (CIEMAT), Madrid, Spain; 113 Universidad Autónoma de Madrid, Madrid, Spain; 114 Instituto Universitario de Ciencias y Tecnologías Espaciales de Asturias (ICTEA), Universidad de Oviedo, Oviedo, Spain; 115 Instituto de Física de Cantabria (IFCA), CSIC-Universidad de Cantabria, Santander, Spain; 116 University of Colombo, Colombo, Sri Lanka; 117 University of Ruhuna, Department of Physics, Matara, Sri Lanka; 118 CERN, European Organization for Nuclear Research, Geneva, Switzerland; 119 Paul Scherrer Institut, Villigen, Switzerland; 120 ETH Zurich-Institute for Particle Physics and Astrophysics (IPA), Zurich, Switzerland; 121 Universität Zürich, Zurich, Switzerland; 122 National Central University, Chung-Li, Taiwan; 123 National Taiwan University (NTU), Taipei, Taiwan; 124 High Energy Physics Research Unit, Department of Physics, Faculty of Science, Chulalongkorn University, Bangkok, Thailand; 125 Çukurova University, Physics Department, Science and Art Faculty, Adana, Turkey; 126 Middle East Technical University, Physics Department, Ankara, Turkey; 127 Bogazici University, Istanbul, Turkey; 128 Istanbul Technical University, Istanbul, Turkey; 129 Istanbul University, Istanbul, Turkey; 130 Institute for Scintillation Materials of National Academy of Science of Ukraine, Kharkiv, Ukraine; 131 National Science Centre, Kharkiv Institute of Physics and Technology, Kharkiv, Ukraine; 132 University of Bristol, Bristol, UK; 133 Rutherford Appleton Laboratory, Didcot, UK; 134 Imperial College, London, UK; 135 Brunel University, Uxbridge, UK; 136 Baylor University, Waco, TX USA; 137 Catholic University of America, Washington, DC USA; 138 The University of Alabama, Tuscaloosa, AL USA; 139 Boston University, Boston, MA USA; 140 Brown University, Providence, RI USA; 141 University of California, Davis, Davis, CA USA; 142 University of California, Los Angeles, CA USA; 143 University of California, Riverside, Riverside, CA USA; 144 University of California, San Diego, La Jolla, CA USA; 145 Department of Physics, University of California, Santa Barbara, Santa Barbara, CA USA; 146 California Institute of Technology, Pasadena, CA USA; 147 Carnegie Mellon University, Pittsburgh, PA USA; 148 University of Colorado Boulder, Boulder, CO USA; 149 Cornell University, Ithaca, NY USA; 150 Fermi National Accelerator Laboratory, Batavia, IL USA; 151 University of Florida, Gainesville, FL USA; 152 Florida State University, Tallahassee, FL USA; 153 Florida Institute of Technology, Melbourne, FL USA; 154 University of Illinois Chicago, Chicago, USA; 155 The University of Iowa, Iowa City, IA USA; 156 Johns Hopkins University, Baltimore, MD USA; 157 The University of Kansas, Lawrence, KS USA; 158 Kansas State University, Manhattan, KS USA; 159 Lawrence Livermore National Laboratory, Livermore, CA USA; 160 University of Maryland, College Park, MD USA; 161 Massachusetts Institute of Technology, Cambridge, MA USA; 162 University of Minnesota, Minneapolis, MN USA; 163 University of Mississippi, Oxford, MS USA; 164 University of Nebraska-Lincoln, Lincoln, NE USA; 165 State University of New York at Buffalo, Buffalo, NY USA; 166 Northeastern University, Boston, MA USA; 167 Northwestern University, Evanston, IL USA; 168 University of Notre Dame, Notre Dame, IN USA; 169 The Ohio State University, Columbus, OH USA; 170 Princeton University, Princeton, NJ USA; 171 University of Puerto Rico, Mayaguez, PR USA; 172 Purdue University, West Lafayette, IN USA; 173 Purdue University Northwest, Hammond, IN USA; 174 Rice University, Houston, TX USA; 175 University of Rochester, Rochester, NY USA; 176 The Rockefeller University, New York, NY USA; 177 Rutgers, The State University of New Jersey, Piscataway, NJ USA; 178 University of Tennessee, Knoxville, TN USA; 179 Texas A&M University, College Station, TX USA; 180 Texas Tech University, Lubbock, TX USA; 181 Vanderbilt University, Nashville, TN USA; 182 University of Virginia, Charlottesville, VA USA; 183 Wayne State University, Detroit, MI USA; 184 University of Wisconsin-Madison, Madison, WI USA; 185 Authors affiliated with an institute or an international laboratory covered by a cooperation agreement with CERN, Geneva, Switzerland; 186 Yerevan State University, Yerevan, Armenia; 187 TU Wien, Vienna, Austria; 188 Institute of Basic and Applied Sciences, Faculty of Engineering, Arab Academy for Science, Technology and Maritime Transport, Alexandria, Egypt; 189 Ghent University, Ghent, Belgium; 190 Universidade Estadual de Campinas, Campinas, Brazil; 191 Federal University of Rio Grande do Sul, Porto Alegre, Brazil; 192 UFMS, Nova Andradina, Brazil; 193 Nanjing Normal University, Nanjing, China; 194 Now at Henan Normal University, Xinxiang, China; 195 Now at The University of Iowa, Iowa City, IA USA; 196 University of Chinese Academy of Sciences, Beijing, China; 197 China Center of Advanced Science and Technology, Beijing, China; 198 University of Chinese Academy of Sciences, Beijing, China; 199 China Spallation Neutron Source, Guangdong, China; 200 Université Libre de Bruxelles, Bruxelles, Belgium; 201 an institute or an international laboratory covered by a cooperation agreement with CERN, Geneva, Switzerland; 202 Cairo University, Cairo, Egypt; 203 Suez University, Suez, Egypt; 204 Now at British University in Egypt, Cairo, Egypt; 205 Birla Institute of Technology, Mesra, Mesra, India; 206 Purdue University, West Lafayette, IN USA; 207 Université de Haute Alsace, Mulhouse, France; 208 Department of Physics, Tsinghua University, Beijing, China; 209 The University of the State of Amazonas, Manaus, Brazil; 210 Erzincan Binali Yildirim University, Erzincan, Turkey; 211 University of Hamburg, Hamburg, Germany; 212 RWTH Aachen University, III. Physikalisches Institut A, Aachen, Germany; 213 Isfahan University of Technology, Isfahan, Iran; 214 Bergische University Wuppertal (BUW), Wuppertal, Germany; 215 Brandenburg University of Technology, Cottbus, Germany; 216 Forschungszentrum Jülich, Juelich, Germany; 217 CERN, European Organization for Nuclear Research, Geneva, Switzerland; 218 Institute of Physics, University of Debrecen, Debrecen, Hungary; 219 Institute of Nuclear Research ATOMKI, Debrecen, Hungary; 220 Now at Universitatea Babes-Bolyai-Facultatea de Fizica, Cluj-Napoca, Romania; 221 Physics Department, Faculty of Science, Assiut University, Assiut, Egypt; 222 HUN-REN Wigner Research Centre for Physics, Budapest, Hungary; 223 Faculty of Informatics, University of Debrecen, Debrecen, Hungary; 224 Punjab Agricultural University, Ludhiana, India; 225 University of Hyderabad, Hyderabad, India; 226 University of Visva-Bharati, Santiniketan, India; 227 Indian Institute of Science (IISc), Bangalore, India; 228 IIT Bhubaneswar, Bhubaneswar, India; 229 Institute of Physics, Bhubaneswar, India; 230 Deutsches Elektronen-Synchrotron, Hamburg, Germany; 231 Department of Physics, Isfahan University of Technology, Isfahan, Iran; 232 Sharif University of Technology, Tehran, Iran; 233 Department of Physics, University of Science and Technology of Mazandaran, Behshahr, Iran; 234 Helwan University, Cairo, Egypt; 235 Italian National Agency for New Technologies, Energy and Sustainable Economic Development, Bologna, Italy; 236 Centro Siciliano di Fisica Nucleare e di Struttura Della Materia, Catania, Italy; 237 Università degli Studi Guglielmo Marconi, Rome, Italy; 238 Scuola Superiore Meridionale, Università di Napoli ’Federico II’, Naples, Italy; 239 Fermi National Accelerator Laboratory, Batavia, IL USA; 240 Università di Napoli ‘Federico II’, Naples, Italy; 241 Ain Shams University, Cairo, Egypt; 242 Consiglio Nazionale delle Ricerche - Istituto Officina dei Materiali, Perugia, Italy; 243 Riga Technical University, Riga, Latvia; 244 Department of Applied Physics, Faculty of Science and Technology, Universiti Kebangsaan Malaysia, Bangi, Malaysia; 245 Consejo Nacional de Ciencia y Tecnología, Mexico City, Mexico; 246 Trincomalee Campus, Eastern University, Sri Lanka, Nilaveli, Sri Lanka; 247 Saegis Campus, Nugegoda, Sri Lanka; 248 INFN Sezione di Pavia, Università di Pavia, Pavia, Italy; 249 National and Kapodistrian University of Athens, Athens, Greece; 250 Ecole Polytechnique Fédérale Lausanne, Lausanne, Switzerland; 251 University of Vienna Faculty of Computer Science, Vienna, Austria; 252 Universität Zürich, Zurich, Switzerland; 253 Stefan Meyer Institute for Subatomic Physics, Vienna, Austria; 254 Laboratoire d’Annecy-le-Vieux de Physique des Particules, IN2P3-CNRS, Annecy-le-Vieux, France; 255 Near East University, Research Center of Experimental Health Science, Mersin, Turkey; 256 Konya Technical University, Konya, Turkey; 257 Izmir Bakircay University, Izmir, Turkey; 258 Adiyaman University, Adiyaman, Turkey; 259 Bozok Universitetesi Rektörlügü, Yozgat, Turkey; 260 Marmara University, Istanbul, Turkey; 261 Milli Savunma University, Istanbul, Turkey; 262 Kafkas University, Kars, Turkey; 263 Now at stanbul Okan University, Istanbul, Turkey; 264 Hacettepe University, Ankara, Turkey; 265 Faculty of Engineering, Istanbul University-Cerrahpasa, Istanbul, Turkey; 266 Yildiz Technical University, Istanbul, Turkey; 267 Vrije Universiteit Brussel, Brussel, Belgium; 268 School of Physics and Astronomy, University of Southampton, Southampton, UK; 269 University of Bristol, Bristol, UK; 270 IPPP Durham University, Durham, UK; 271 Monash University, Faculty of Science, Clayton, Australia; 272 Now at an institute or an international laboratory covered by a cooperation agreement with CERN, Geneva, Switzerland; 273 Università di Torino, Turin, Italy; 274 Bethel University, St. Paul, MN USA; 275 Karamanoğlu Mehmetbey University, Karaman, Turkey; 276 California Institute of Technology, Pasadena, CA USA; 277 United States Naval Academy, Annapolis, MD USA; 278 Bingol University, Bingol, Turkey; 279 Georgian Technical University, Tbilisi, Georgia; 280 Sinop University, Sinop, Turkey; 281 Erciyes University, Kayseri, Turkey; 282 Horia Hulubei National Institute of Physics and Nuclear Engineering (IFIN-HH), Bucharest, Romania; 283 Texas A&M University at Qatar, Doha, Qatar; 284 Kyungpook National University, Daegu, Korea; 285 another institute or international laboratory covered by a cooperation agreement with CERN, Geneva, Switzerland; 286 Universiteit Antwerpen, Antwerpen, Belgium; 287 Yerevan Physics Institute, Yerevan, Armenia; 288 Northeastern University, Boston, MA USA; 289 Imperial College, London, UK; 290 Institute of Nuclear Physics of the Uzbekistan Academy of Sciences, Tashkent, Uzbekistan; 291 CERN, 1211 Geneva 23, Switzerland

## Abstract

Using proton–proton collision data corresponding to an integrated luminosity of $140\hbox { fb}^{-1}$ collected by the CMS experiment at $\sqrt{s}= 13\,\text {Te}\hspace{-.08em}\text {V}$, the ${{{\Lambda }} _{\text {b}}^{{0}}} \rightarrow {{\text {J}/\uppsi }} {{{\Xi }} ^{{-}}} {{\text {K}} ^{{+}}}$ decay is observed for the first time, with a statistical significance exceeding 5 standard deviations. The relative branching fraction, with respect to the ${{{\Lambda }} _{\text {b}}^{{0}}} \rightarrow {{{\uppsi }} ({2\textrm{S}})} {{\Lambda }}$ decay, is measured to be $\mathcal {B}({{{\Lambda }} _{\text {b}}^{{0}}} \rightarrow {{\text {J}/\uppsi }} {{{\Xi }} ^{{-}}} {{\text {K}} ^{{+}}} )/\mathcal {B}({{{\Lambda }} _{\text {b}}^{{0}}} \rightarrow {{{\uppsi }} ({2\textrm{S}})} {{\Lambda }} ) = [3.38\pm 1.02\pm 0.61\pm 0.03]\%$, where the first uncertainty is statistical, the second is systematic, and the third is related to the uncertainties in $\mathcal {B}({{{\uppsi }} ({2\textrm{S}})} \rightarrow {{\text {J}/\uppsi }} {{{\uppi }} ^{{+}}} {{{\uppi }} ^{{-}}} )$ and $\mathcal {B}({{{\Xi }} ^{{-}}} \rightarrow {{\Lambda }} {{{\uppi }} ^{{-}}} )$.

## Introduction

Multibody decays of beauty hadrons present a rich laboratory to search for intermediate resonances in the decay products. When decay products contain a charmonium state, such intermediate resonances could decay into a charmonium meson and a hadron, which could be a manifestation of their exotic nature. An important turning point in exotic spectroscopy was achieved at the LHC, when the LHCb Collaboration reported the observation of statistically significant ${{\text {J}/\uppsi }} {{\text {p}}}$ pentaquark-like structures in the decay of the lightest beauty baryon ${{{\Lambda }} _{\text {b}}^{{0}}} \rightarrow {{\text {J}/\uppsi }} {{\text {p}}} {{{\text {K}}}^{{-}}}$ [1]. Various interpretations of these structures have been proposed [2, 3], including tightly bound hidden-charm $[]$ pentaquark states [4, 5], loosely bound molecular baryon-meson states [6–8], or being due to a double triangle singularity [9]. More recently, additional exotic states have been reported by LHCb in the decays ${{{\Lambda }} _{\text {b}}^{{0}}} \rightarrow {{\text {J}/\uppsi }} {{\text {p}}} {{{\text {K}}}^{{-}}}$  [10], ${{{\Xi }} _{\text {b}}^{{-}}} \rightarrow {{\text {J}/\uppsi }} {{\Lambda }} {{{\text {K}}}^{{-}}}$ [11],  [12], and ${{{\text {B}}}^{{-}}} \rightarrow {{\text {J}/\uppsi }} {{\Lambda }} {{{{\text {p}}}}}$ [13]. Up to now, the hidden-charm pentaquark candidates have been reported only in ${{\text {J}/\uppsi }} {{\text {p}}}$ and ${{\text {J}/\uppsi }} {{\Lambda }}$ systems. Investigation of other channels with heavier baryons in the decay products, such as ${{{\Xi }} ^{{-}}}$ and ${{{\Omega }} ^{{-}}}$, could unveil the existence of doubly or triply strange pentaquarks [14, 15].

In this paper, we report on the search for the ${{{\Lambda }} _{\text {b}}^{{0}}} \rightarrow {{\text {J}/\uppsi }} {{{\Xi }} ^{{-}}} {{\text {K}} ^{{+}}}$ decay, where the ${{\text {J}/\uppsi }} \rightarrow {{{\upmu }} ^{{+}}} {{{\upmu }} ^{{-}}}$, ${{{\Xi }} ^{{-}}} \rightarrow {{\Lambda }} {{{\uppi }} ^{{-}}}$, and ${{\Lambda }} \rightarrow {{\text {p}}} {{{\uppi }} ^{{-}}}$ channels are used to reconstruct the intermediate decay products. Charge-conjugate states are implied throughout the text. The measurement of the ratio of branching fractions

1 $$\begin{aligned} \begin{aligned} \mathcal {R}&\equiv \frac{\mathcal {B}({{{\Lambda }} _{\text {b}}^{{0}}} \rightarrow {{\text {J}/\uppsi }} {{{\Xi }} ^{{-}}} {{\text {K}} ^{{+}}} )}{\mathcal {B}({{{\Lambda }} _{\text {b}}^{{0}}} \rightarrow {{{\uppsi }} ({2\textrm{S}})} {{\Lambda }} )} = \frac{N({{{\Lambda }} _{\text {b}}^{{0}}} \rightarrow {{\text {J}/\uppsi }} {{{\Xi }} ^{{-}}} {{\text {K}} ^{{+}}} )}{N({{{\Lambda }} _{\text {b}}^{{0}}} \rightarrow {{{\uppsi }} ({2\textrm{S}})} {{\Lambda }} )} \\&\quad \times \frac{\epsilon _{{{{\uppsi }} ({2\textrm{S}})} {{\Lambda }} }}{\epsilon _{{{\text {J}/\uppsi }} {{{\Xi }} ^{{-}}} {{\text {K}} ^{{+}}} }} \,\frac{\mathcal {B}({{{\uppsi }} ({2\textrm{S}})} \rightarrow {{\text {J}/\uppsi }} {{{\uppi }} ^{{+}}} {{{\uppi }} ^{{-}}} )}{\mathcal {B}({{{\Xi }} ^{{-}}} \rightarrow {{\Lambda }} {{{\uppi }} ^{{-}}} )} \end{aligned} \end{aligned}$$

is also reported, where *N* is the measured ${{{\Lambda }} _{\text {b}}^{{0}}}$ yield and $\epsilon$ is the total efficiency. The normalization channel is chosen to be ${{{\Lambda }} _{\text {b}}^{{0}}} \rightarrow {{{\uppsi }} ({2\textrm{S}})} {{\Lambda }}$, with the subsequent ${{{\uppsi }} ({2\textrm{S}})} \rightarrow {{\text {J}/\uppsi }} {{{\uppi }} ^{{+}}} {{{\uppi }} ^{{-}}}$ and ${{\text {J}/\uppsi }} \rightarrow {{{\upmu }} ^{{+}}} {{{\upmu }} ^{{-}}}$ decays, because of its similar decay topology and kinematics to the signal decay, leading to the reduction of many systematic uncertainties. The branching fractions of the intermediate decays $\mathcal {B}({{\text {J}/\uppsi }} \rightarrow {{{\upmu }} ^{{+}}} {{{\upmu }} ^{{-}}} )$ and $\mathcal {B}({{\Lambda }} \rightarrow {{\text {p}}} {{{\uppi }} ^{{-}}} )$ cancel in the ratio. Invariant mass distributions of the three two-body combinations for the signal channel are also presented in order to look for intermediate resonances.

The analysis uses proton–proton (pp) collision data recorded by the CMS experiment in 2016–2018, at $\sqrt{s}=13\,\text {Te}\hspace{-.08em}\text {V}$, corresponding to an integrated luminosity of $140\hbox { fb}^{-1}$ [16–18]. Tabulated results are provided in the HEPData record for this analysis [19].

## The CMS detector and simulated event samples

The central feature of the CMS apparatus is a superconducting solenoid of 6 m internal diameter, providing a magnetic field of 3.8 T. Within the solenoid volume are a silicon pixel and strip tracker, a lead tungstate crystal electromagnetic calorimeter, and a brass and scintillator hadron calorimeter, each composed of a barrel and two endcap sections. Forward calorimeters extend the pseudorapidity coverage provided by the barrel and endcap detectors. Muons are measured in gas-ionization detectors embedded in the steel flux-return yoke outside the solenoid. A more detailed description of the CMS detector, together with a definition of the coordinate system used and the relevant kinematic variables, can be found in Ref. [20].

Muons are measured in the pseudorapidity range $|{\eta }| < 2.4$, with detection planes made using three technologies: drift tubes, cathode strip chambers, and resistive-plate chambers. Matching muons to tracks measured in the silicon tracker results in a transverse momentum ($p_{\textrm{T}}$) resolution for muons with $p_{\textrm{T}}$ up to 100$\,\text {Ge}\hspace{-.08em}\text {V}$ of 1% in the barrel and 3% in the endcaps. The silicon tracker used in 2016 measured charged particles within the range $|{\eta }| < 2.5$. For nonisolated particles of $1< p_{\textrm{T}} < 10\,\text {Ge}\hspace{-.08em}\text {V}$ and $|{\eta }| < 1.4$, the track resolutions were typically 1.5% in $p_{\textrm{T}}$ and 25–90 $\,\mu \text {m}$ in the transverse impact parameter [21]. At the start of 2017, a new pixel detector was installed [22]; the upgraded tracker measured nonisolated particles of $1< p_{\textrm{T}} < 10\,\text {Ge}\hspace{-.08em}\text {V}$ up to $|{\eta }| < 3$ with typical resolutions of 1.5% in $p_{\textrm{T}}$ and 20–75$\,\mu \text {m}$ in the transverse impact parameter [23].

Events of interest are selected using a two-tiered trigger system [24]. The first level, composed of custom hardware processors, uses information from the calorimeters and muon detectors to select events at a rate of around 100 kHz within a fixed latency of about 4$\,\upmu \text {s}$  [25]. The second level, known as the high-level trigger (HLT), consists of a farm of computing processors running a version of the full event reconstruction software optimized for fast processing, and reduces the event rate to around 1 kHz before data storage. All events used in this analysis are selected by a set of triggers requiring two identified muons of opposite charge plus an additional track to form a secondary vertex, displaced from the region of the pp interactions. The trigger demanded for each muon to have $p_{\textrm{T}} >4\,\text {Ge}\hspace{-.08em}\text {V}$ and to pass within 2 cm of the beam axis. The dimuon system was required to have $p_{\textrm{T}} >6.9\,\text {Ge}\hspace{-.08em}\text {V}$, invariant mass between 2.9 and 3.3$\,\text {Ge}\hspace{-.08em}\text {V}$, a vertex fit probability greater than 10%, a separation of the secondary vertex relative to the beam axis in the transverse plane larger than 3 standard deviations (s.d.), and a cosine of the angle in the transverse plane between the dimuon momentum vector and the vector joining the beam axis and the dimuon vertex greater than 0.9. The additional track was required to have $p_{\textrm{T}} >0.8\,(1.2)\,\text {Ge}\hspace{-.08em}\text {V}$ and an impact parameter with respect to the beam axis greater than 0 (2) s.d., for data collected in 2016 (2017–2018). Finally, the two muons and the additional track were required to originate from the same vertex with a $\chi ^2$ per degree of freedom (dof) less than 10.

Monte Carlo (MC) simulated event samples are generated with Pythia v8.240 [26] using the CP5 underlying event tune [27]. The evtgen 1.6.0 [28] program models the beauty baryon decays ${{{\Lambda }} _{\text {b}}^{{0}}} \rightarrow {{\text {J}/\uppsi }} {{{\Xi }} ^{{-}}} {{\text {K}} ^{{+}}}$ and ${{{\Lambda }} _{\text {b}}^{{0}}} \rightarrow {{{\uppsi }} ({2\textrm{S}})} {{\Lambda }}$ with a phase space decay model, followed by the ${{{\uppsi }} ({2\textrm{S}})} \rightarrow {{\text {J}/\uppsi }} {{{\uppi }} ^{{+}}} {{{\uppi }} ^{{-}}}$ and ${{\text {J}/\uppsi }} \rightarrow {{{\upmu }} ^{{+}}} {{{\upmu }} ^{{-}}}$ decays. Final-state radiation is included in evtgen using photos 3.61 [29]. The events are then passed through a detailed Geant4-based simulation [30] of the CMS detector, including also the decays of long-lived hyperons ${{{\Xi }} ^{{-}}} \rightarrow {{\Lambda }} {{{\uppi }} ^{{-}}}$ and ${{\Lambda }} \rightarrow {{\text {p}}} {{{\uppi }} ^{{-}}}$, followed by the trigger and reconstruction algorithms identical to those used for the collision data. The simulation includes additional interactions due to multiple pp collisions in each bunch crossing, with the same distribution as observed in the experiment.

## Event reconstruction and selection

The reconstruction for all the decays considered in this analysis starts by finding two muons of opposite charge, which must match those that triggered the event readout and pass the soft-muon identification criteria [31]. The offline selection for both muons requires $p_{\textrm{T}} ({{{\upmu }} ^{{\pm }}}) >3\,\text {Ge}\hspace{-.08em}\text {V}$, $|\eta ({{{\upmu }} ^{{\pm }}})|<2.4$, $\chi ^2$ fit probability to a common dimuon vertex $P_{\text {vtx}} ({{{\upmu }} ^{{+}}} {{{\upmu }} ^{{-}}} ) > 1\%$, and dimuon invariant mass $2.9<m({{{\upmu }} ^{{+}}} {{{\upmu }} ^{{-}}} )<3.3\,\text {Ge}\hspace{-.08em}\text {V}$.

The ${{\Lambda }} \rightarrow {{\text {p}}} {{{\uppi }} ^{{-}}}$ candidates are selected from displaced two-prong vertices as described in Ref. [32]. The track with the higher momentum is assumed to be the proton one, and together with the pion track it is fit to a common vertex with their invariant mass constrained to the known ${{\Lambda }}$ hyperon mass of $m_{\textrm{PDG}}({{\Lambda }} ) = 1115.683\,\text {Me}\hspace{-.08em}\text {V}$ [33]. The $\chi ^2$ fit probability for the ${{\Lambda }}$ vertex is required to be $P_{\text {vtx}} ({{\text {p}}} {{{\uppi }} ^{{-}}} ) > 1\%$.

For the signal channel, to form the ${{{\Xi }} ^{{-}}} \rightarrow {{\Lambda }} {{{\uppi }} ^{{-}}}$ candidates, an additional high-purity [21] track assumed to be a pion is selected with $p_{\textrm{T}} >0.2\,\text {Ge}\hspace{-.08em}\text {V}$. This track and the selected ${{\Lambda }}$ candidate are then fit to a common vertex with the ${{\Lambda }} {{{\uppi }} ^{{-}}}$ mass constrained to the known ${{{\Xi }} ^{{-}}}$ hyperon mass of $m_{\textrm{PDG}}({{{\Xi }} ^{{-}}} ) = 1321.71$ $\,\text {Me}\hspace{-.08em}\text {V}$  [33]. To form the ${{{\Lambda }} _{\text {b}}^{{0}}} \rightarrow {{\text {J}/\uppsi }} {{{\Xi }} ^{{-}}} {{\text {K}} ^{{+}}}$ candidate, a high-purity track is chosen with an assigned kaon mass and $p_{\textrm{T}} ({{\text {K}} ^{{+}}})>1.2\,\text {Ge}\hspace{-.08em}\text {V}$, which aligns with the HLT $p_{\textrm{T}}$ requirement. The final reconstruction step in the signal channel is the ${{{\upmu }} ^{{+}}} {{{\upmu }} ^{{-}}} {{{\Xi }} ^{{-}}} {{\text {K}} ^{{+}}}$ vertex fit with a $\chi ^2$ probability above 1%, where the dimuon mass is constrained to the world-average ${{\text {J}/\uppsi }}$ meson mass of $3096.9\,\text {Me}\hspace{-.08em}\text {V}$ [33].

For the normalization channel, two high-purity tracks of opposite charges with $p_{\textrm{T}} >0.4\,\text {Ge}\hspace{-.08em}\text {V}$, assumed to be pions from the ${{{\uppsi }} ({2\textrm{S}})} \rightarrow {{\text {J}/\uppsi }} {{{\uppi }} ^{{+}}} {{{\uppi }} ^{{-}}}$ decay, are selected. One of them is required to have $p_{\textrm{T}} >1.2\,\text {Ge}\hspace{-.08em}\text {V}$ to match the HLT $p_{\textrm{T}}$ requirement. The ${{{\Lambda }} _{\text {b}}^{{0}}}$ candidates are obtained by a vertex fit of the ${{{\upmu }} ^{{+}}} {{{\upmu }} ^{{-}}} {{{\uppi }} ^{{+}}} {{{\uppi }} ^{{-}}} {{\Lambda }}$ system with a ${{\text {J}/\uppsi }} \rightarrow {{{\upmu }} ^{{+}}} {{{\upmu }} ^{{-}}}$ mass constraint, as for the signal channel. The invariant mass of the ${{\text {J}/\uppsi }} {{{\uppi }} ^{{+}}} {{{\uppi }} ^{{-}}}$ candidates is required to be in the range $3.60<m({{\text {J}/\uppsi }} {{{\uppi }} ^{{+}}} {{{\uppi }} ^{{-}}} )<3.95\,\text {Ge}\hspace{-.08em}\text {V}$.

From all reconstructed pp collision points in each event, the primary vertex (PV) is chosen as the one with the smallest ${{{\Lambda }} _{\text {b}}^{{0}}}$ pointing angle, which is the angle between the momentum of the ${{{\Lambda }} _{\text {b}}^{{0}}}$ candidate and the vector from the PV to the reconstructed ${{{\Lambda }} _{\text {b}}^{{0}}}$ candidate vertex. If any of the tracks used in the ${{{\Lambda }} _{\text {b}}^{{0}}}$ candidate reconstruction were included in the fit of the chosen PV, they are removed, and the PV is refitted.

Selection criteria for the signal channel ${{{\Lambda }} _{\text {b}}^{{0}}} \rightarrow {{\text {J}/\uppsi }} {{{\Xi }} ^{{-}}} {{\text {K}} ^{{+}}}$ are optimized using the Punzi figure of merit [34]. The signal efficiency is evaluated using simulated event samples. Estimation of the background yield involves combining the collision data from the ${{{\Lambda }} _{\text {b}}^{{0}}}$ mass sideband, excluding the signal region which spans twice the mass resolution around the known ${{{\Lambda }} _{\text {b}}^{{0}}}$ mass. Additionally, the wrong-sign candidates (${{\text {J}/\uppsi }} {{{\Xi }} ^{{-}}} {{{\text {K}}}^{{-}}}$ and ${{\text {J}/\uppsi }} \bar{\Xi }^+{{\text {K}} ^{{+}}}$) from the full mass range are included, after ensuring that the mass distribution of the wrong-sign candidates matches that of the correct-sign ones. Combining these two background sources reduces the impact of the statistical uncertainty in the optimization procedure. The variables used in the optimization include the $p_{\textrm{T}}$ of all decay products; the flight length significance in the transverse plane of the ${{{\Lambda }} _{\text {b}}^{{0}}}$, ${{\Lambda }}$, and ${{{\Xi }} ^{{-}}}$ baryon candidates and the corresponding pointing angles; the impact parameter significance with respect to the PV in the transverse plane for the tracks; the vertex fit probabilities; and the mass windows for hyperon candidates. The order of the cuts is determined randomly, and in several rounds of optimization this order was different each time; all rounds have converged to the same final set of optimized cuts. The resulting criteria are summarized in Table 1. The background is reduced by a factor of 15 after the optimization, whereas the signal efficiency is 70% of the initial selection described above. The selection criteria in the normalization channel ${{{\Lambda }} _{\text {b}}^{{0}}} \rightarrow {{{\uppsi }} ({2\textrm{S}})} {{\Lambda }}$ are chosen to be the same, wherever possible, as in the signal channel, to reduce the systematic uncertainties. The ${{\text {J}/\uppsi }} {{{\uppi }} ^{{+}}} {{{\uppi }} ^{{-}}}$ mass is required to be within 11.1$\,\text {Me}\hspace{-.08em}\text {V}$ of the known ${{{\uppsi }} ({2\textrm{S}})}$ meson mass of $3686.1\,\text {Me}\hspace{-.08em}\text {V}$ [33], which corresponds to approximately 2.5 times the mass resolution.

For the measurement of $\mathcal {R}$ defined in Eq. (1), the pion from the ${{{\Xi }} ^{{-}}}$ decay is required to have $p_{\textrm{T}} >0.4\,\text {Ge}\hspace{-.08em}\text {V}$. Additionally, the HLT requirements are repeated offline by requiring $p_{\textrm{T}} (\mu )>4\,\text {Ge}\hspace{-.08em}\text {V}$, $p_{\textrm{T}} ({{\text {J}/\uppsi }} ) >6.9\,\text {Ge}\hspace{-.08em}\text {V}$, $P_{\text {vtx}} ({{{\upmu }} ^{{+}}} {{{\upmu }} ^{{-}}} )>5\%$, and track (kaon for the signal channel, the harder of the two pions in the normalization channel) impact parameter above 2 s.d. with respect to the PV. These extra criteria ensure that events from potentially inadequately modeled phase space regions are avoided, as the reliability of the efficiency evaluation from simulated samples in those regions is questionable. Nevertheless, the reconstruction algorithm works reliably in those regions, and thus the corresponding events are used to study the mass distribution, as discussed in the following section.

In less than 5% of the events, multiple ${{{\Lambda }} _{\text {b}}^{{0}}}$ candidates in the same channel are found. The rate is consistent in both channels and all candidates are used in the analysis. Selecting a single candidate has a negligible effect on the results.

**Table 1:** Optimized selection criteria for the signal decay mode ${{{\Lambda }} _{\text {b}}^{{0}}} \rightarrow {{\text {J}/\uppsi }} {{{\Xi }} ^{{-}}} {{\text {K}} ^{{+}}}$. The first two requirements are applied using the momenta before the corresponding mass constraint

Variable	Selection
$|{m({{\text {p}}} {{{\uppi }} ^{{-}}} )-m_{\textrm{PDG}}({{\Lambda }} )}|$	<8 MeV
$|{m({{\Lambda }} {{{\uppi }} ^{{-}}} )-m_{\textrm{PDG}}({{{\Xi }} ^{{-}}} )}|$	<6 MeV
$p_{\textrm{T}} ({{{\Lambda }} _{\text {b}}^{{0}}} )$	>11.5 GeV
$p_{\textrm{T}} ({{\text {J}/\uppsi }} )$	>6.5 GeV
$p_{\textrm{T}} ({{{\Xi }} ^{{-}}} )$	>2.6 GeV
$p_{\textrm{T}} ({{\Lambda }} )$	>2.2 GeV
$p_{\textrm{T}} ({{\text {K}} ^{{+}}})$	>1.2 GeV
${{{\upmu }} ^{{+}}} {{{\upmu }} ^{{-}}} {{{\Xi }} ^{{-}}} {{\text {K}} ^{{+}}}$ vertex fit probability	>5%
${{\Lambda }} {{{\uppi }} ^{{-}}}$ vertex fit probability	>5%
${{\text {p}}} {{{\uppi }} ^{{-}}}$ vertex fit probability	>1%
${{{\Xi }} ^{{-}}}$ vertex displacement from ${{{\Lambda }} _{\text {b}}^{{0}}}$ vertex	>3 s.d.
${{\Lambda }}$ vertex displacement from ${{{\Xi }} ^{{-}}}$ vertex	>0 s.d.
${{{\Lambda }} _{\text {b}}^{{0}}}$ vertex displacement from PV	>3 s.d.
Angle between ${{{\Xi }} ^{{-}}}$ momentum and displacement	<0.0447 rad
Angle between ${{\Lambda }}$ momentum and displacement	<0.14 rad
Angle between ${{{\Lambda }} _{\text {b}}^{{0}}}$ momentum and displacement	<0.0447 rad
PV impact parameter for pion from ${{{\Xi }} ^{{-}}}$ decay	>0.4 s.d.
PV impact parameter for kaon	>0.4 s.d.

## Invariant mass distributions

**Fig. 1:**
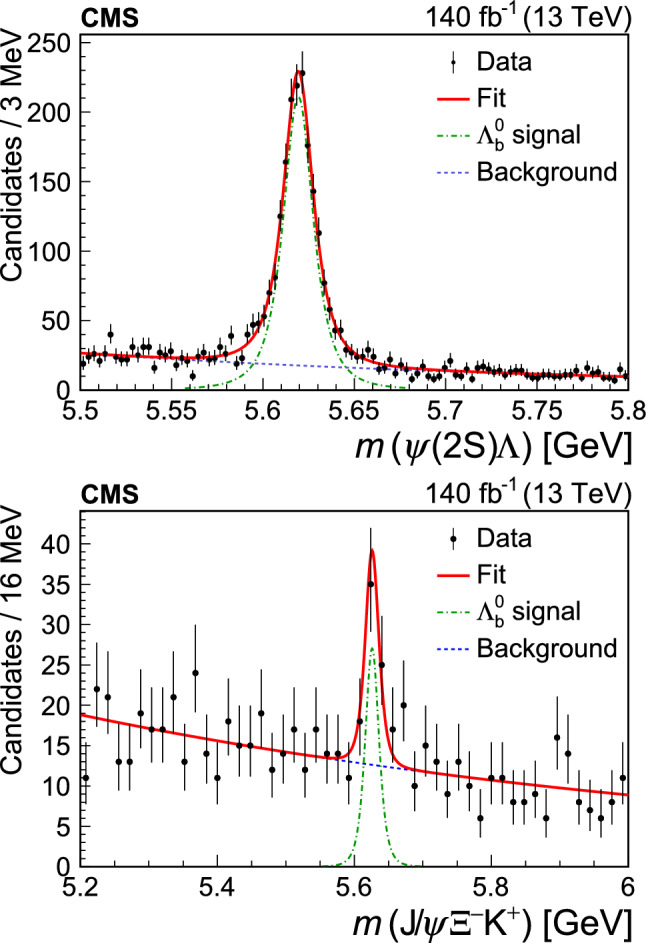
Measured ${{{\uppsi }} ({2\textrm{S}})} {{\Lambda }}$ (upper) and ${{\text {J}/\uppsi }} {{{\Xi }} ^{{-}}} {{\text {K}} ^{{+}}}$ (lower) invariant mass distributions and overlaid fit results

The measured mass distribution of the ${{{\uppsi }} ({2\textrm{S}})} {{\Lambda }}$ candidates is shown in Fig. 1 (left) together with the results of an unbinned maximum likelihood fit. The signal is modeled with a Student’s *t*-distribution [35] with all parameters (mean, $\sigma$, *n*) free. The combinatorial background is described by an exponential function with a free slope parameter and normalization. The fitted mass of $5619.3\pm 0.3\,\text {Me}\hspace{-.08em}\text {V}$ is in agreement with the world-average ${{{\Lambda }} _{\text {b}}^{{0}}}$ mass of $5619.60\pm 0.17\,\text {Me}\hspace{-.08em}\text {V}$ [33], and the mass resolution of $8.90\pm 0.40\,\text {Me}\hspace{-.08em}\text {V}$ is slightly larger than, yet in agreement with, its value of 8.52$\,\text {Me}\hspace{-.08em}\text {V}$ found in simulation. The measured yield is $N({{{\Lambda }} _{\text {b}}^{{0}}} \rightarrow {{{\uppsi }} ({2\textrm{S}})} {{\Lambda }} )=1744\pm 63$. The $\chi ^2$ between the binned distribution and the fit function is 76.6 for 94 degrees of freedom, demonstrating the good quality of the fit.

The measured invariant mass distribution of the selected ${{\text {J}/\uppsi }} {{{\Xi }} ^{{-}}} {{\text {K}} ^{{+}}}$ candidates is shown in Fig. 1 (lower). A narrow peak at the ${{{\Lambda }} _{\text {b}}^{{0}}}$ mass is seen on top of a smooth background. The ${{{\Lambda }} _{\text {b}}^{{0}}}$ signal is modeled with a Student’s *t*-distribution with mean and $\sigma$ floating, but the *n* parameter fixed to the value found by fitting the simulated distribution, because of the limited signal yield of $N({{{\Lambda }} _{\text {b}}^{{0}}} \rightarrow {{\text {J}/\uppsi }} {{{\Xi }} ^{{-}}} {{\text {K}} ^{{+}}} )=46\pm 11$. The background is fitted with an exponential function. The ${{{\Lambda }} _{\text {b}}^{{0}}}$ mass returned by the fit ($5625.9 \pm 3.2\,\text {Me}\hspace{-.08em}\text {V}$) is within 2 s.d. of the world-average value [33]. The width of the signal peak ($\sigma$) is found to be $10.4\pm 3.3\,\text {Me}\hspace{-.08em}\text {V}$, consistent within 1.2 s.d. with the value found in simulation, $6.6\pm 0.2\,\text {Me}\hspace{-.08em}\text {V}$. The fit quality is good, as demonstrated by the $\chi ^2/\textrm{dof}=30.1/45$ for the binned distribution.

The signal significance is evaluated using the likelihood ratio technique by applying the background-only and signal-plus-background hypotheses. In these two fits, a Gaussian constraint is applied on the background shape parameter to the one obtained from a fit to the wrong-sign data. Similarly, a Gaussian constraint is applied to the signal shape parameter *n* (from simulation) and the resolution $\sigma =\sigma _{\textrm{MC}}\, (8.90/8.52)$. The correction factor is extracted from the normalization channel and accounts for the difference in the widths of the peak between the measured and simulated event samples. The mean value of the peak is also Gaussian-constrained with a central value and uncertainty equal to the known ${{{\Lambda }} _{\text {b}}^{{0}}}$ mass and its uncertainty [33], respectively. The fit with the signal-plus-background model with these constraints returns a signal yield of $36\pm 8$ and is presented in Appendix A. Since the conditions of Wilks’ theorem [36] are satisfied, the asymptotic formulae of Ref. [37] (Eqs. (12) and (52)) are used to determine the ${{{\Lambda }} _{\text {b}}^{{0}}} \rightarrow {{\text {J}/\uppsi }} {{{\Xi }} ^{{-}}} {{\text {K}} ^{{+}}}$ signal significance, which is found to be 5.8 standard deviations. To evaluate the effect of the choice of the model for fitting the signal significance, several alternative models of signal and background were tested, including double-Gaussian or Johnson [38] functions for the signal and a second-degree polynomial or a modified threshold function for the background. An alternative without a constraint on the background shape was also tested. The significance obtained with the alternative models varies in the range from 5.3 to 5.9 standard deviations. This allows us to claim the first observation of the ${{{\Lambda }} _{\text {b}}^{{0}}} \rightarrow {{\text {J}/\uppsi }} {{{\Xi }} ^{{-}}} {{\text {K}} ^{{+}}}$ decay.

**Fig. 2:**
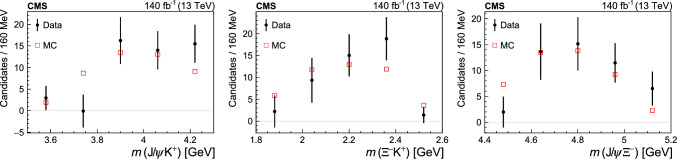
Intermediate invariant mass distributions of the ${{{\Lambda }} _{\text {b}}^{{0}}} \rightarrow {{\text {J}/\uppsi }} {{{\Xi }} ^{{-}}} {{\text {K}} ^{{+}}}$ decay. The filled circles and empty squares show the measured background-subtracted distributions and the results from the simulation with a phase-space model, respectively

The sensitivity of this analysis to potential pentaquark signals in the intermediate invariant mass distributions of the ${{{\Lambda }} _{\text {b}}^{{0}}} \rightarrow {{\text {J}/\uppsi }} {{{\Xi }} ^{{-}}} {{\text {K}} ^{{+}}}$ decay is limited by the low signal yield. The background-subtracted two-body invariant mass distributions, obtained with the $_\mathrm {{s}}\mathcal {P}\textrm{lot}$ technique [39], are shown in Fig. 2. The distributions do not show any clear peaks and agree, within uncertainties, with the predictions from the phase space simulation. The distributions are also consistent with the results of extracting the yields by fitting the ${{{\Lambda }} _{\text {b}}^{{0}}}$ signal in each of the five intermediate invariant mass bins.

**Fig. 3:**
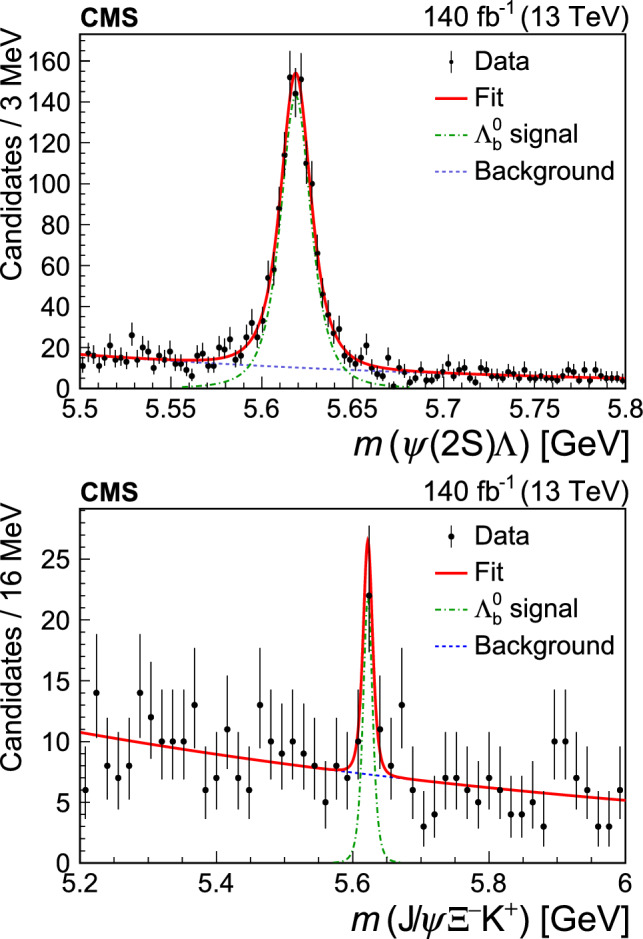
Measured ${{{\uppsi }} ({2\textrm{S}})} {{\Lambda }}$ (upper) and ${{\text {J}/\uppsi }} {{{\Xi }} ^{{-}}} {{\text {K}} ^{{+}}}$ (lower) invariant mass distributions and corresponding fits used for the measurement of $\mathcal {R}$

For the measurement of $\mathcal {R}$ (Eq. (1)), more stringent requirements are used, as explained at the end of Sect. 3, and the measured signal yields decrease to $1179\pm 47$ and $23\pm 7$ for the ${{{\Lambda }} _{\text {b}}^{{0}}} \rightarrow {{{\uppsi }} ({2\textrm{S}})} {{\Lambda }}$ and ${{{\Lambda }} _{\text {b}}^{{0}}} \rightarrow {{\text {J}/\uppsi }} {{{\Xi }} ^{{-}}} {{\text {K}} ^{{+}}}$ channels, respectively, using unconstrained fits as for Fig. 1. These are the baseline results referred to in Sect. 6. The corresponding mass distributions and fits are presented in Fig. 3.

## Efficiencies

Efficiencies for the signal and normalization channels are calculated using simulated event samples. The total efficiency is calculated by factorizing into two components: detector acceptance and a combined trigger, reconstruction, and selection efficiency.

As only the ratio of the total efficiencies is needed to measure $\mathcal {R}$, the systematic uncertainties associated with the muon and track reconstruction are reduced. The obtained efficiency ratio is $\epsilon _{{{{\uppsi }} ({2\textrm{S}})} {{\Lambda }} }/\epsilon _{{{\text {J}/\uppsi }} {{{\Xi }} ^{{-}}} {{\text {K}} ^{{+}}} } = 5.06\pm 0.29$, where the uncertainty reflects the limited size of the simulated samples. Efficiencies for different years of data-taking are combined with weights corresponding to the integrated luminosity collected in each year. The efficiency for the ${{{\Lambda }} _{\text {b}}^{{0}}} \rightarrow {{\text {J}/\uppsi }} {{{\Xi }} ^{{-}}} {{\text {K}} ^{{+}}}$ channel is significantly lower than that for the ${{{\Lambda }} _{\text {b}}^{{0}}} \rightarrow {{{\uppsi }} ({2\textrm{S}})} {{\Lambda }}$ channel for several reasons including the low energy release in the ${{{\Xi }} ^{{-}}} \rightarrow {{\Lambda }} {{{\uppi }} ^{{-}}}$ decay, resulting in a low-momentum pion track.

## Systematic uncertainties

Many systematic uncertainties, related to the muon reconstruction and identification as well as to the trigger efficiency, partially cancel in the measured ratios. Since the signal and normalization channels have the same number of tracks in the final state, most uncertainties related to track reconstruction also cancel in the measured ratio $\mathcal {R}$. However, the $p_{\textrm{T}}$ spectrum of kaons from the ${{{\Lambda }} _{\text {b}}^{{0}}} \rightarrow {{\text {J}/\uppsi }} {{{\Xi }} ^{{-}}} {{\text {K}} ^{{+}}}$ decay is observed to differ from that of the highest-$p_{\textrm{T}}$ pion in the ${{{\Lambda }} _{\text {b}}^{{0}}} \rightarrow {{{\uppsi }} ({2\textrm{S}})} {{\Lambda }}$ channel used for normalization. Despite the signal and normalization channels having the same number of final-state tracks, an uncertainty of 2.3% [40] is included, which reflects the difference in tracking efficiency between the measured and simulated event samples. The MC event samples are validated using the normalization channel by comparing the measured distributions of variables used in the event selection, after background subtraction, to those found in simulation; no significant discrepancies are found in most of the distributions. A small discrepancy was observed in the $p_{\textrm{T}} ({{{\Lambda }} _{\text {b}}^{{0}}} )$ distribution, and the MC event samples for both channels were reweighted using $p_{\textrm{T}} ({{{\Lambda }} _{\text {b}}^{{0}}} )$-dependent weights so that the $p_{\textrm{T}} ({{{\Lambda }} _{\text {b}}^{{0}}} )$ distribution in the weighted simulation sample matches the background-subtracted distribution measured in the ${{{\Lambda }} _{\text {b}}^{{0}}} \rightarrow {{{\uppsi }} ({2\textrm{S}})} {{\Lambda }}$ channel. The efficiency ratio evaluated using these weighted MC samples is found to be $\epsilon _{{{{\uppsi }} ({2\textrm{S}})} {{\Lambda }} }/\epsilon _{{{\text {J}/\uppsi }} {{{\Xi }} ^{{-}}} {{\text {K}} ^{{+}}} }=4.82\pm 0.39$, which is lower by 4.7%, yet still in agreement with the value reported in the previous section. An uncertainty of 4.7% is assigned to account for potential mismodeling of the $p_{\textrm{T}} ({{{\Lambda }} _{\text {b}}^{{0}}} )$ spectrum.

The systematic uncertainty related to the choice of the signal model is evaluated by testing three different models. For the normalization channel ${{{\Lambda }} _{\text {b}}^{{0}}} \rightarrow {{{\uppsi }} ({2\textrm{S}})} {{\Lambda }}$ the signal shape parameters are floating, while for the signal channel ${{{\Lambda }} _{\text {b}}^{{0}}} \rightarrow {{\text {J}/\uppsi }} {{{\Xi }} ^{{-}}} {{\text {K}} ^{{+}}}$ the mass resolution parameters are fixed to those found in simulation, after correcting the width of the peak for the ratio between the two resolutions in the measured and simulated event samples evaluated in the normalization channel. The tested models simultaneously vary the signal and normalization channels and use a Student’s *t*-distribution, a double-Gaussian, and a Johnson function [38] to model the signal. The largest deviation in the ratio of the ${{{\Lambda }} _{\text {b}}^{{0}}}$ signal yields from the baseline value is taken as the systematic uncertainty.

The systematic uncertainty related to the choice of the background model is estimated in a similar way, with three alternative models: a second-degree polynomial, a threshold function [41, 42] multiplied by an exponential, and a threshold function multiplied by a first-degree polynomial.

By requiring the ${{\text {J}/\uppsi }} {{{\uppi }} ^{{+}}} {{{\uppi }} ^{{-}}}$ invariant mass to be near the ${{{\uppsi }} ({2\textrm{S}})}$ mass, we aim to select ${{{\Lambda }} _{\text {b}}^{{0}}} \rightarrow {{{\uppsi }} ({2\textrm{S}})} {{\Lambda }}$ decays. Nevertheless, other ${{{\Lambda }} _{\text {b}}^{{0}}} \rightarrow {{\text {J}/\uppsi }} {{{\uppi }} ^{{+}}} {{{\uppi }} ^{{-}}} \Lambda$ decays, either from different intermediate resonances or from four-body nonresonant decays may contribute. To estimate this contribution, we use the $_\mathrm {{s}}\mathcal {P}\textrm{lot}$ technique to subtract the background under the ${{{\Lambda }} _{\text {b}}^{{0}}}$ peak and plot the ${{\text {J}/\uppsi }} {{{\uppi }} ^{{+}}} {{{\uppi }} ^{{-}}}$ mass in an expanded mass region. The ${{\text {J}/\uppsi }} {{{\uppi }} ^{{+}}} {{{\uppi }} ^{{-}}}$ mass is fitted with a signal component for the ${{{\uppsi }} ({2\textrm{S}})}$ and a background component for everything else. The integral of the background over the range used to select ${{{\uppsi }} ({2\textrm{S}})}$ events yields 30 events, which is 2.5% of the total yield (1179 events). This value is used as the systematic uncertainty related to non-${{{\uppsi }} ({2\textrm{S}})}$ contributions in the normalization channel.

The uncertainty in the efficiency ratio due to the limited size of the simulated samples, calculated to be 5.6% in Sect. 5, is considered as a systematic uncertainty.

In order to assess the reliability of the efficiency evaluation from the simulated samples, the selection criteria on muon and ${{\text {J}/\uppsi }}$ $p_{\textrm{T}}$, dimuon vertex probability, track impact parameter, and $p_{\textrm{T}}$ of the soft pion from ${{{\Xi }} ^{{-}}}$ decay are tightened, one at a time, until the signal efficiency decreases by 10 or 20% with respect to that obtained with the selection used for the $\mathcal {R}$ measurement. The analysis is repeated each time, and the value of $\mathcal {R}$ is re-calculated and compared to the baseline $\mathcal {R}$ value. The differences (*d*) between the two values and their uncertainties ($\delta d$), which also account for the correlation between the two values, are evaluated. The largest value of $\sqrt{\smash [b]{d^2-(\delta d)^2}}$ among the different variations of the selection criteria is found to be 14.3% and is used as the systematic uncertainty in the efficiency ratio.

Table 2 summarizes the previously discussed systematic uncertainties in the ratio $\mathcal {R}$. The total uncertainty is calculated as the sum in quadrature of the individual sources.

**Table 2:** The relative systematic uncertainties in the measurement of $\mathcal {R}$

Source	Uncertainty (%)
Tracking efficiency	2.3
$p_{\textrm{T}} ({{{\Lambda }} _{\text {b}}^{{0}}} )$ spectrum	4.7
Signal model	3.9
Background model	6.7
Non-${{{\uppsi }} ({2\textrm{S}})}$ contribution	2.5
Limited size of MC samples	5.6
Selection efficiency	14.3
Total	18.2

## Branching fraction ratio measurement

The branching fraction of the newly observed ${{{\Lambda }} _{\text {b}}^{{0}}} \rightarrow {{\text {J}/\uppsi }} {{{\Xi }} ^{{-}}} {{\text {K}} ^{{+}}}$ decay, with respect to the ${{{\Lambda }} _{\text {b}}^{{0}}} \rightarrow {{{\uppsi }} ({2\textrm{S}})} {{\Lambda }}$ one, is measured using Eq. (1) to be

$$\begin{aligned}\begin{aligned} \mathcal {R}&\equiv \frac{\mathcal {B}({{{\Lambda }} _{\text {b}}^{{0}}} \rightarrow {{\text {J}/\uppsi }} {{{\Xi }} ^{{-}}} {{\text {K}} ^{{+}}} )}{\mathcal {B}({{{\Lambda }} _{\text {b}}^{{0}}} \rightarrow {{{\uppsi }} ({2\textrm{S}})} {{\Lambda }} )} \\&= [3.38\pm 1.02\,\text {(stat)} \pm 0.61\,\text {(syst)} \pm 0.03\,(\mathcal {B}) ]\%, \end{aligned} \end{aligned}$$

where the last uncertainty is related to the uncertainties in the branching fractions $\mathcal {B}({{{\uppsi }} ({2\textrm{S}})} \rightarrow {{\text {J}/\uppsi }} {{{\uppi }} ^{{+}}} {{{\uppi }} ^{{-}}} )=34.68\pm 0.30\%$ and $\mathcal {B}({{{\Xi }} ^{{-}}} \rightarrow {{\Lambda }} {{{\uppi }} ^{{-}}} )=99.887\pm 0.035\%$ [33].

## Summary

The ${{{\Lambda }} _{\text {b}}^{{0}}} \rightarrow {{\text {J}/\uppsi }} {{{\Xi }} ^{{-}}} {{\text {K}} ^{{+}}}$ decay is observed with a significance exceeding 5 standard deviations using $\sqrt{s}=13\,\text {Te}\hspace{-.08em}\text {V}$ proton–proton collision data corresponding to an integrated luminosity of $140\hbox { fb}^{-1}$ collected by the CMS experiment. The branching fraction is measured with respect to the ${{{\Lambda }} _{\text {b}}^{{0}}} \rightarrow {{{\uppsi }} ({2\textrm{S}})} {{\Lambda }}$ decay to be $\mathcal {B}({{{\Lambda }} _{\text {b}}^{{0}}} \rightarrow {{\text {J}/\uppsi }} {{{\Xi }} ^{{-}}} {{\text {K}} ^{{+}}} )/\mathcal {B}({{{\Lambda }} _{\text {b}}^{{0}}} \rightarrow {{{\uppsi }} ({2\textrm{S}})} {{\Lambda }} ) = [3.38\pm 1.02\,\text {(stat)} \pm 0.61\,\text {(syst)} \pm 0.03\,(\mathcal {B}) ]\%$. The distributions of intermediate invariant masses $m({{\text {J}/\uppsi }} {{{\Xi }} ^{{-}}} )$, $m({{\text {J}/\uppsi }} {{\text {K}} ^{{+}}})$, and $m({{{\Xi }} ^{{-}}} {{\text {K}} ^{{+}}})$ from the ${{{\Lambda }} _{\text {b}}^{{0}}} \rightarrow {{\text {J}/\uppsi }} {{{\Xi }} ^{{-}}} {{\text {K}} ^{{+}}}$ decay are also presented. This is the first discovered multibody decay containing the ${{\text {J}/\uppsi }} {{{\Xi }} ^{{-}}}$ system, which opens the possibility to search for doubly-strange hidden-charm pentaquarks when more data are collected. The new results are important for understanding the strong interaction processes in hadronic decays of beauty baryons and the possible formation of exotic multiquark states.

## Figures and Tables

**Fig. 4:**
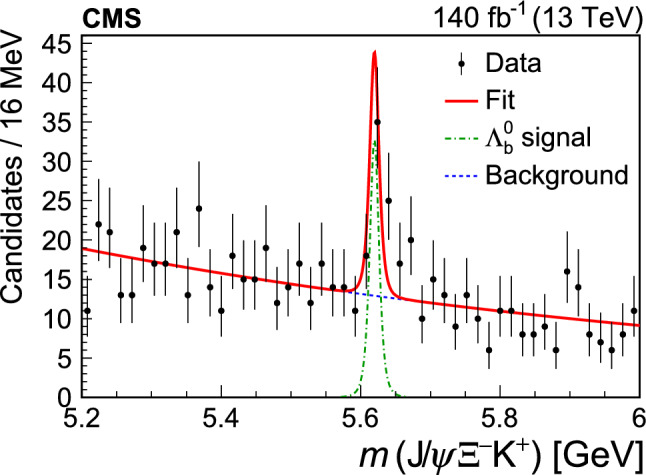
Measured ${{\text {J}/\uppsi }} {{{\Xi }} ^{{-}}} {{\text {K}} ^{{+}}}$ invariant mass distribution and overlaid constrained fit result

## Data Availability

This manuscript has associated data in a data repository. [Author’s comment: Some data used for this analysis may be made available. Almost certainly not all data will be released near term.]

## Invariant mass distribution fit with constraints

The fit of the measured ${{\text {J}/\uppsi }} {{{\Xi }} ^{{-}}} {{\text {K}} ^{{+}}}$ invariant mass distribution with constraints on the background shape parameter, signal shape parameter, resolution, and the mean value of the peak is presented in Fig. 4. The fit quality is good, as demonstrated by $\chi ^2/\textrm{dof}=35.6/44$ for the binned distribution.

## Code Availability Statement

Code/software cannot be made available for reasons disclosed in the code availability statement. [Author’s comment: Some software (the core software) is available. Not all code used is available.]
